# QSAR-Co-X: an open source toolkit for multitarget QSAR modelling

**DOI:** 10.1186/s13321-021-00508-0

**Published:** 2021-04-15

**Authors:** Amit Kumar Halder, M. Natália Dias Soeiro Cordeiro

**Affiliations:** LAQV@REQUIMTE/Faculty of Sciences, University of Porto, 4169-007 Porto, Portugal

**Keywords:** QSAR, Multitarget models, Software tools, Feature selection, Machine learning

## Abstract

Quantitative structure activity relationships (QSAR) modelling is a well-known computational tool, often used in a wide variety of applications. Yet one of the major drawbacks of conventional QSAR modelling is that models are set up based on a limited number of experimental and/or theoretical conditions. To overcome this, the so-called multitasking or multitarget QSAR (mt-QSAR) approaches have emerged as new computational tools able to integrate diverse chemical and biological data into a *single* model equation, thus extending and improving the reliability of this type of modelling. We have developed *QSAR-Co-X*, an open source python–based toolkit (available to download at https://github.com/ncordeirfcup/QSAR-Co-X) for supporting mt-QSAR modelling following the Box-Jenkins moving average approach. The new toolkit embodies several functionalities for dataset selection and curation plus computation of descriptors, for setting up linear and non-linear models, as well as for a comprehensive results analysis. The workflow within this toolkit is guided by a cohort of multiple statistical parameters and graphical outputs onwards assessing both the predictivity and the robustness of the derived mt-QSAR models. To monitor and demonstrate the functionalities of the designed toolkit, four case-studies pertaining to previously reported datasets are examined here. We believe that this new toolkit, along with our previously launched *QSAR-Co* code, will significantly contribute to make mt-QSAR modelling widely and routinely applicable.

**Supplementary Information:**

The online version contains supplementary material available at 10.1186/s13321-021-00508-0.

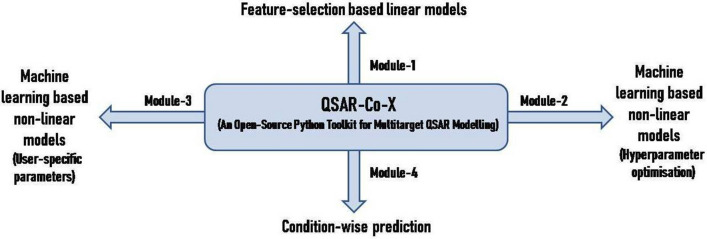

## Introduction

Quantitative Structure–Activity Relationships (QSAR) modelling is one of the most frequently employed in silico techniques for chemical data mining and analysis. Though QSAR has been introduced more than 50 years ago, it remains as an efficient technique for building mathematical models to find out crucial structural requirement for targeting specific response variables (*e*.*g.*, activity, toxicity, physicochemical properties, etc.). At the same time, QSAR provides one of the most effective strategies for predicting properties of new chemicals and also for identifying potential hits through virtual screening of chemical libraries [1, 2]. The last few decades have witnessed several transformations in the field of QSAR modelling, owing to the progress in model development strategies, data mining techniques, validation methodologies, along with machine learning and statistical analysis tools [3, 4]. Nevertheless, the quest for new modelling strategies is still ongoing to further improve the overall efficacy of QSAR modelling [1, 5, 6]. For example, one of the major limitations of conventional QSAR is that models are developed for the response variable(s), regardless of the experimental (or theoretical) conditions followed to obtain such response variable(s). In reality however, the researchers come across data-points pertaining to various experimental and/or theoretical conditions, the inclusion of which may significantly improve the scope of QSAR modelling. This has paved the way to unconventional computational modelling approaches, so-called multitasking, or multitarget QSAR (mt-QSAR), which are able to integrate data under different conditions into a *single* model equation for simultaneous prediction of the targeted response variable(s) [7–9]. Therefore, the interest of QSAR practitioner researchers over such mt-modelling has been growing steadily [1, 5]. In particular, mt-QSAR modelling techniques based on the Box-Jenkins moving average approach have already proved to be highly efficient in dealing with datasets pertaining to multiple conditions [10–14]. Our group has recently developed an open source standalone software “QSAR-Co” (https://sites.google.com/view/qsar-co) [15] to set up classification-based QSAR models. Briefly, *QSAR-Co* enables users to set up linear or non-linear classification models, by resorting to the Genetic Algorithm based Linear Discriminant Analysis (GA-LDA) [16, 17] or to the Random Forests (RF) [18] classifier, respectively. As *per* our experience so far, mt-QSAR modelling is highly sensitive to the strategies used for model development especially because the number of starting descriptors increases depending on the number of experimental (and/or theoretical) conditions. The possibility of employing a larger range of development strategies will definitely improve the usefulness and scope of such mt-QSAR modelling. The present work moves a step forward and describes a new toolkit named *QSAR-Co-X*, which apart from supporting the development of multitarget QSAR models based on the Box-Jenkins moving average approach, allows the usage of various descriptor generation schemes, along with several model development strategies, feature selection algorithms and machine learning tools, as well as model selection and validation methodologies. As it will be seen, the *QSAR-Co-X* software implements a number of additional utilities that renders a much more compact and well-designed platform for multitarget QSAR modelling, following the principles of QSAR modelling recommended by the OECD (Organization for Economic Cooperation and Development) [19]. The major differences between these two software tools are listed and commented in Table 1.

**Table 1:** Major differences between QSAR-Co and QSAR-Co-X

No	Utility	QSAR-Co	QSAR-Co-X	Remarks
1	Feature selection	One (GA)	Two (FS and SFS)	–
2	Reproducibility of linear modelling	Low	High	Given the same sample size and number of descriptors, GA produces different LDA models on different runs, whereas both the FS and SFS always yield the same model
3	Diagnosis of intercollinearity among variables	Not available	Available and automatically performed	Very helpful for ascertaining the robustness of the derived linear models
4	Dataset division options	Random, Kennard-Stone, Euclidean-based	Random, pre-defined, *k*-MCA	Since only the random division option is fast, the other QSAR-Co options were replaced to reduce computational time
5	Automatic generation of the validation set	Not available	Available	Unlike QSAR-Co, QSAR-Co-X allows generating both the screening and validation sets
6	Statistical parameters for the validation set	Manual calculations are required	Automatic calculation	Automatic calculation allows fast selection of the models
7	Number of Box-Jenkins operators available	One (pre-defined)	Four (three pre-defined and one user-specific)	Additional and more flexible operators were added to QSAR-Co-X
8	*Y*_*c*_ randomisation	Not available	Available	A modified form of the *Y*-randomisation technique that incorporates the influence of experimental elements
9	Machine-learning tools	One (RF only)	Six (*k*NN, SVM, RF, NB, GB, and MLP)	QSAR-Co-X affords several non-linear modelling tools
10	Number of parameters that may be altered in RF modelling	5	8	QSAR-Co-X offers more flexibility for setting up RF models
11	Comparative analysis of multiple ML methods	Not possible	Possible	Useful to decide which ML method performs best
12	Hyperparameter tuning options for ML methods	Not available	Available	Extremely useful to find optimised non-linear models
13	User specific parameter settings for building non-linear models	For RF only	For kNN, SVM, RF, NB, GB, and MLP	–
14	Display of ROC plots (linear modelling)	For sub-training and test sets	For sub-training, test and validation sets	–
15	Condition-wise prediction	Not available	Available	Useful to understand how the developed model performs against individual experimental conditions, particularly for large datasets

As can be seen, two additional feature selection techniques were included for establishing LDA models, namely fast-stepwise (FS) and sequential forward selection (SFS). Even though the GA implemented earlier in *QSAR-Co* has proved to be a highly efficient feature selection technique, judging from our previous analyses [11, 20], the implementation of these additional feature selection techniques in *QSAR-Co-X* improves the scope of LDA modelling in multiple ways. Firstly, the application of more feature selection techniques enhances the chances of obtaining more predictive models especially for big data analysis [21]. Secondly, the GA selection involves the random generation of an initial population, which usually requires several runs to produce the most statistically significant (or optimised) model. Also, due to this randomisation step, the models generated by GA-LDA lack reproducibility. As such, both FS and SFS techniques are more straightforward and reproducible, allowing the swift establishment of linear discriminant models. Finally, simultaneous application of GA with the two newly implemented feature selection algorithms can help finding a greater number of LDA models, thereby increasing the possibility of consensus modelling. Additionally, the *QSAR-Co-X* software provides significant modifications as far as strategies for the development of non-linear models are concerned. First of all, it comprises a toolkit for building non-linear models by resorting to six different machine learning (ML) algorithms. One of its modules assists in tuning hyperparameters of such ML tools (not included in *QSAR-Co* [15]) for achieving optimised models. As an alternative, a separate module is available for setting up user-specific parameters meant to a rapidly development of non-linear models. Alike *QSAR-Co*, model development in *QSAR-Co-X* is guided by descriptor pre-treatment, two-stage external validation, and determination of the applicability domain of linear and non-linear models. Still the *QSAR-Co-X*’ toolkit applies additional options for calculating the modified descriptors using different types of the Box-Jenkins moving average operators. It also provides a modified *Y*-based randomisation method [15], so-called *Y*_*c*_-randomisation, to check the robustness of the derived linear model. The latter may be used for ‘condition-wise prediction’ in which the user may check its predictivity for each experimental/theoretical condition. The relevance of whole these new utilities implemented in the toolkit are exemplified with four case studies.

## Implementation

The *QSAR-Co-X* version 1.0.0 is an open source standalone toolkit developed using Python 3 [22]. It can be downloaded freely from https://github.com/ncordeirfcup/QSAR-Co-X. The manual provided along with the toolkit describes in detail its operating procedures. The *QSAR-Co-X* toolkit comprises four modules, namely: (i) LM (abbreviation for linear modelling); (ii) NLG (abbreviation for non-linear modelling with grid search); (iii) NLU (abbreviation for non-linear modelling with user specific parameters); and (iv) CWP (abbreviation for condition-wise prediction). Details about the functionalities of each of these modules are described below.

### Module 1 (LM)

**Fig. 1:**
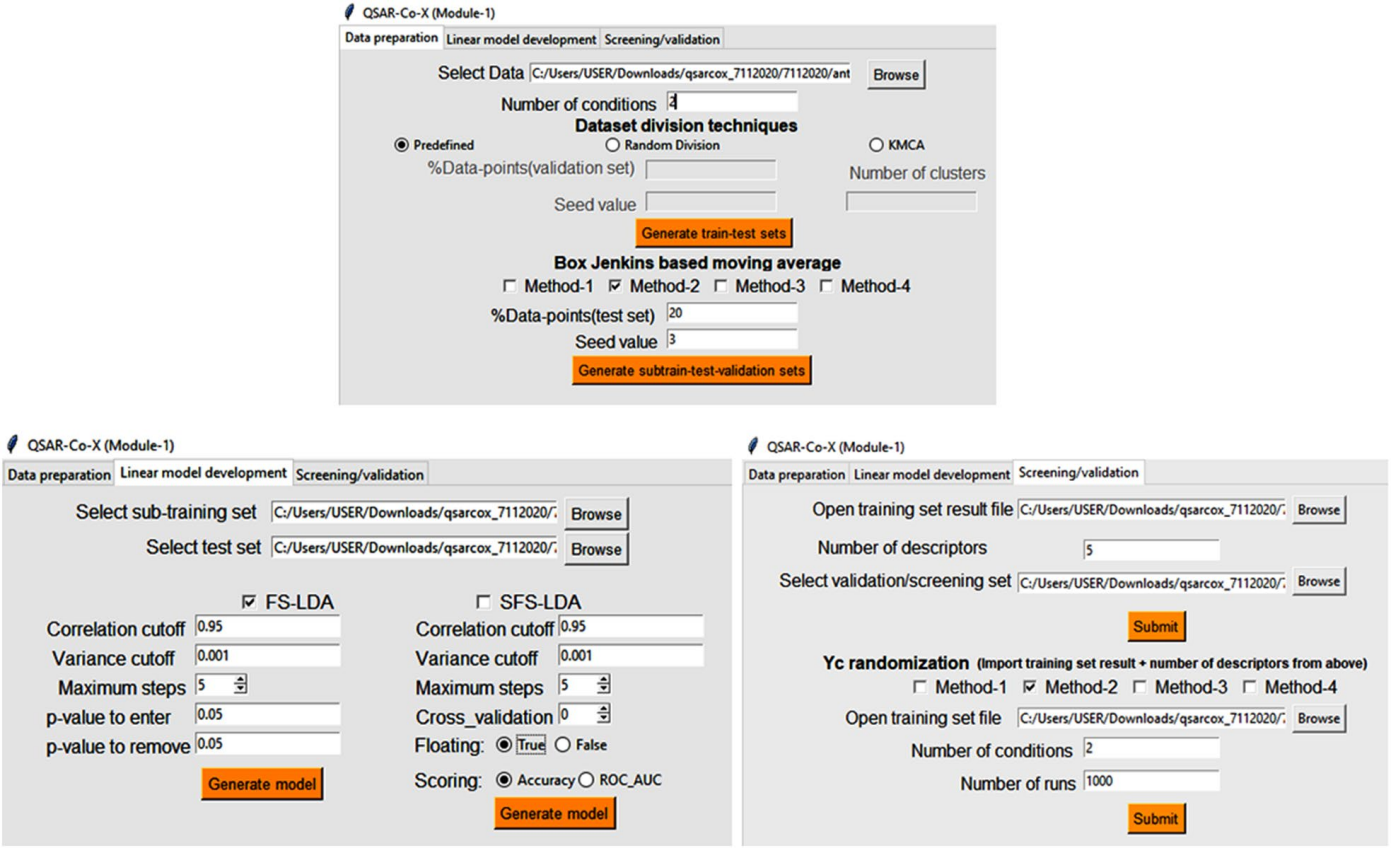
Screenshot of the Module1 graphic user interface from toolkit QSAR-Co-X

This module assists in dataset division, the calculation of deviation descriptors from input descriptors using the Box-Jenkins scheme and data pre-treatment. Along with these, the module comprises two feature selection algorithms for development and validation of the LDA models (see the screenshot in Fig. 1). The following sixth-step procedure is adopted for establishing the linear models.

#### Step 1-Dataset division

The first step of any mt-QSAR model encompasses a division of the initial dataset into a training and a validation set. In this module, that may be performed following three schemes, namely: (a) pre-determined data distribution, (b) random division and (c) *k*-means cluster analysis (*k*MCA) based data division [20]. In the first scheme (a), the user is allowed to explicitly provide information about the training and validation set samples, *i.e*., the set samples are to be tagged as ‘Train’ and ‘Test’, respectively. This is extremely important when the user intends to compare a model with a specific data-distribution previously derived from any other in silico tool with the models developed using *QSAR-Co-X*. In the second scheme (b), the random division of the dataset is obtained on the basis of the user-specific percentage of validation set data-points. At the same time, different training and validation sets may be obtained by changing the random seed values. As an alternative to random data-splitting, the user may opt for a *k*-Means Cluster Analysis-based rational dataset division strategy (*k*MCA) [20, 23]. In the latter option, the dataset is first divided into *n* (user specific) clusters on the basis of input descriptors. Subsequently, a specific number of validations set samples are randomly collected from each cluster. Similar to the random division scheme, the ratio between the training and validation sets may be varied and, simultaneously, different combinations of these sets obtained by changing the random seed value. The python code *KMCA.py* included in the toolkit allows performing the *k*MCA-based dataset division.

#### Step 2-box−jenkins moving average approach

The most important part of current mt-QSAR modelling is the calculation of the deviation descriptors from the input descriptors, following the Box-Jenkins moving average approach. The input descriptors can be calculated using any commercial or non-commercial software packages (e.g.: DRAGON [24] or QuBiLS-MAS [25]) but then these have to be modified to incorporate the influence of different experimental (and/or theoretical) elements ($c_{j}$).

The mathematical details of the Box-Jenkins moving average approach have been extensively described in the past [8, 9, 26], so we will restrict ourselves to a short description highlighting only its most important aspects. There are different ways for calculating the modified descriptors by this approach, the simplest one being as follows:

1 $${\Delta (}D_{i} {)}\,c_{j} = D_{i} - avg\;{(}D_{i} {)}\,c_{j}$$

Specifically, the new descriptors $\Delta {(}D_{i} {)}c_{j}$ are calculated by the difference between the input descriptors of the active chemicals (*D*_*i*_) and their averages $avg\;(D_{i} )c_{j}$ − *i.e.* their arithmetic mean for a specific element of the experimental and/or theoretical conditions (ontology) $c_{j}$ [8]:

2 $$avg\;{(}D_{i} {)}c_{j} = \sum\limits_{i = 1}^{{n{(}c_{j} {)}}} {D_{i} {/}n{(}c_{j} {)}}$$

In recent years, different forms for these modified descriptors have however been suggested depending on the conditions. For example, the descriptors may be standardised by resorting to the maximum ($D_{i\max }$) and minimum ($D_{i\min }$) values of input descriptors [12]:

3 $${\Delta (}D_{i} {)}c_{j} = \frac{{D_{i} - avg\,{(}D_{i} {)}c_{j} }}{{D_{i\max } - D_{i\min } }}$$

Analogously, the elements of $c_{j}$ may be also standardised, as recently proposed by Speck-Planche [27], leading to the following expression for the modified descriptors:

4 $$\Delta {(}D_{i} {)}c_{j} = \frac{{D_{i} - avg{(}D_{i} {)}c_{j} }}{{{(}D_{i\max } - D_{i\min } {)}\,p{(}c_{j} {)}_{c} }}$$

In this equation $p{(}c_{j} {)}$ represents the a priori probability of finding the datapoints pertaining to particular conditions and so, $p{(}c_{j} {)}_{c}$ may simply be obtained by dividing the number of actives in the data under a specific element of $c_{j}$−$n{(}c_{j} {)}$−by the total number of datapoints *N* (see Eq. 5). More details about this topic will be discussed within the case study 3 reported in this work.

5 $$p{(}c_{j} {)}_{c} = \frac{{n\left( {c_{j} } \right)}}{N}$$

In the present toolkit, the user can choose one of the four methods provided (Method1-4) to compute the modified descriptors. The first three ones are based on Eqs. 1, 3 and 4, respectively. Note that both Method2 and Method3 do not work with invariant descriptors and that may hamper further calculations. Therefore, in these two methods, a descriptor pre-treatment is carried to remove constant descriptors. Finally, Method4 allows the user to apply its own proper scheme for establishing the $p{(}c_{j} {)}$ values [27, 28], and the resulting modified descriptors are thus represented as follows:

6 $$\Delta {(}D_{i} {)}c_{j} = \frac{{D_{i} - avg{(}D_{i} {)}c_{j} }}{{p{(}c_{j} {)}_{u} }}$$

where the term $p{(}c_{j} {)}_{u}$ denotes the user-specific $p{(}c_{j} {)}$, whose values should be provided as inputs. Within that context, the $p{(}c_{j} {)}$ values do not need to be always calculated since these may also be obtained from experimental and/or theoretical data. As an example, in a previous study [26], $p{(}c_{j} {)}$ accounted for the degree of reliability of the experimental information and the values of 0.55, 0.75 and 1.00 were used for the data-points, which were classified as ‘auto-curation’, ‘intermediate’ and ‘expert’ according to the labelling of the CHEMBL database, respectively.

Similar to *QSAR-Co*, the current toolkit uses two stages of external validation for mt-QSAR modelling, thereby requiring two separate test sets as well. As mentioned earlier, the dataset is initially split into training and validation sets by employing pre-defined sets, random division or *k*MCA-based systematic division schemes. The Box-Jenkins moving average approach is then applied to calculate the modified descriptors for the training set, by selecting one of the methods described above. The training set and their corresponding modified descriptors are subsequently randomly sub-divided into a sub-training and a test set (or calibration set). Here, it is important to remark that the $avg{(}D_{i} {)}c_{j}$ values obtained from the training set are applied to calculate the modified descriptors for the validation set and thus, the latter can be recognised as the ‘ideal test set’ due to the fact that its data-points do not participate either in the model development or in the descriptor calculation. On the other hand, the test set may be employed both as a ‘calibration set’ (especially for GA-LDA) and as an ‘external validation set’.

#### Step 3-Data pre-treatment

The user specific data pre-treatment step of this module includes: (a) removal of highly correlated descriptors based on the user specified correlation cut-off, and (b) removal of the descriptors with less variation based also on the user specified variation cut-off. What is more, constant descriptors fail to produce models for all feature selection procedures.

#### Step 4-Linear model development

Two feature selection algorithms are used for setting up the linear discriminant analysis (LDA) models, namely: (a) fast stepwise (FS) and (b) sequential stepwise (SFS). Although many feature selection algorithms are available, the two chosen here can be highly efficient while handling mt-QSAR modelling because of their ability to fast generate models. Both these can be employed along with the GA selection, which is available in *QSAR-Co*, but that requires many iterations for finding the optimised LDA models. FS is a very popular algorithm in which the independent descriptors are included in the model stepwise depending on the specific statistical parameter *p*-value, and it has previously been successfully employed to set up mt-QSAR models [10, 26]. The usual criteria for forward selection (*i.e*., *p*-value to enter) and backward elimination (*p*-value to remove) are set in the present toolkit. This is, the descriptor with the lowest *p*-value is included first and subsequently other descriptors are included in the model based on the lowest *p*-value only if the criteria for forward selection are met. Yet, if the *p*-value of a descriptor included in the model is found to be greater than ‘*p*-value to remove’, it is eliminated from the model. The SFS algorithm adds features into an empty set until the performance of the model is not improved either by addition of another feature or the maximum number of features is reached [29]. Similar to FS, it is also a greedy search algorithm where the best subsets of descriptors are selected stepwise and the model performance is judged by the user specific statistical parameters, denoted as ‘scoring’ parameters. In the current version of QSAR-Co-X, two scoring parameters are provided, namely: ‘Accuracy’ and ‘AUROC’ (see description below). The users may develop separate models by varying these two scoring parameters in QSAR-Co-X (see Case Study 4 for more details).

In contrast to GA, in which the generation of models is based on a randomisation process, these two feature selection algorithms for LDA are systematic and therefore faster. In this work, we resorted to the tool *SequentialFeatureSelector* from the library mlxtend (version 0.17.1: http://rasbt.github.io/mlxtend/) for developing the FS-/SFS-LDA models. In both, the *singular value decomposition* (svd), recommended for data containing large number of features is applied within the Scikit-learn Linear Discriminant Analysis package [30, 31].

#### Step 5-model validation

The reliability and statistical significance of the models are evaluated by goodness-of-fit as well as by internal and external validation criteria.

Goodness-of-fit for the sub-training set is assessed by looking at the usual *p* and *F* (Fisher’s statistics) parameters along with the Wilks’ lambda (λ) statistic [32]. The latter essentially measures the discriminatory power of the LDA classification models, *i.e.*, how well they separate cases into groups. It is equal to the proportion of the total variance in the discriminant scores not explained by differences among groups, and can take values from zero, perfect discrimination, to one, no discrimination. Similar to Wilk’s λ, the *F*-test measures how better a complex model is in comparison to a simpler version of the same model in itscapacity to explain the variance in the response variable [33].

All these statistical parameters are calculated with the help of the “Statsmodel” ordinary least square python library (https://www.statsmodels.org/stable/api.html/).

The overall predictivity of the models is checked by examining the confusion matrix, which includes the number of true positive (TP), true negative (TN), false positive (FP) and false negative (FN) samples. Simultaneously based on those numbers, other statistical parameters such as the Sensitivity, Specificity, Accuracy, F1-score, and the Matthew correlation coefficient (MCC) are computed for the sub-training, test and validation sets (see Eq. 7), as well as the area under the receiver operating characteristic curve (AUROC) [34–36]. Additionally, the ROC curves are automatically created for each model.

$${\text{Sensitivity}} = {\text{TP/(TP}} + {\text{FN)}}$$

$${\text{Specificity}} = {\text{TN/(TN}} + {\text{FP)}}$$

$${\text{Accuracy}} = {\text{(TP}} + {\text{TN)/(TP}} + {\text{TN}} + {\text{FP}} + {\text{FN)}}$$

$${\text{F1}} - {\text{score}} = {\text{2TP/(2TP}} + {\text{FP}} + {\text{FN)}}$$

7 $${\text{MCC}} = \frac{{{\text{TP}} \cdot {\text{TN}} - {\text{FP}} \cdot {\text{FN}}}}{{\sqrt {{\text{[(TP}} + {\text{FP)(TP}} + {\text{FN)(TN}} + {\text{FP)(TN}} + {\text{FN)]}}} }}$$

Apart from confirming the internal and external predictivity, the choice of the best linear model should be guided through additional criteria. For example, highly correlated descriptors in the linear model may reduce its overall significance and therefore, the degree of collinearity among its descriptors must be carefully examined. To do so, the current module automatically generates the cross-correlation matrix for the selected sub-training set descriptors. It is also important to assess the applicability domain (AD) of the derived model−*i.e.,* the response and chemical structure space within which the model makes reliable predictions. Here, the models’ AD is estimated by the *standardisation approach* as proposed earlier by Roy et al. [37], allowing as well to identify possible structural chemical outliers. The python code for this approach is provided in the *applicability.py* file of the toolkit.

#### Step 6-*Y*_*c*_-randomisation

In the previous *QSAR-Co* [15], the *Y*-randomization scheme has been implemented to judge the performance of the derived linear models. That is, following a classical scheme, the statistical quality in data description of the original linear model is compared to that of models generated upon randomly shuffling several times the response variable based upon the user specified ‘number of runs’−*n*. Since in the Box-Jenkins based mt-QSAR modelling, the experimental/theoretical conditions elements participate in the determination of modified descriptors, the *Y*-randomization is slightly modified here and named *Y*_*c*_-randomization−*i.e., Y* randomization with conditions. In this new scheme, along with the response variables, the experimental elements $c_{j}$ are also scrambled *n* times, and thus *n* randomised data-matrices being generated. The several models are subsequently rederived with these randomised data and averages and the Wilks’ lambda (*λ*_*r*_) and accuracy (*Accuracy*_*r*_) calculated. In a robust model, the values obtained for these two parameters should be considerably less than Wilks’ λ and accuracy of the original model. The phyton code *ycr.py* tackles this scheme in *QSAR-Co-X*.

### Module 2 (NLG)−hyperparameter tuning

**Fig. 2:**
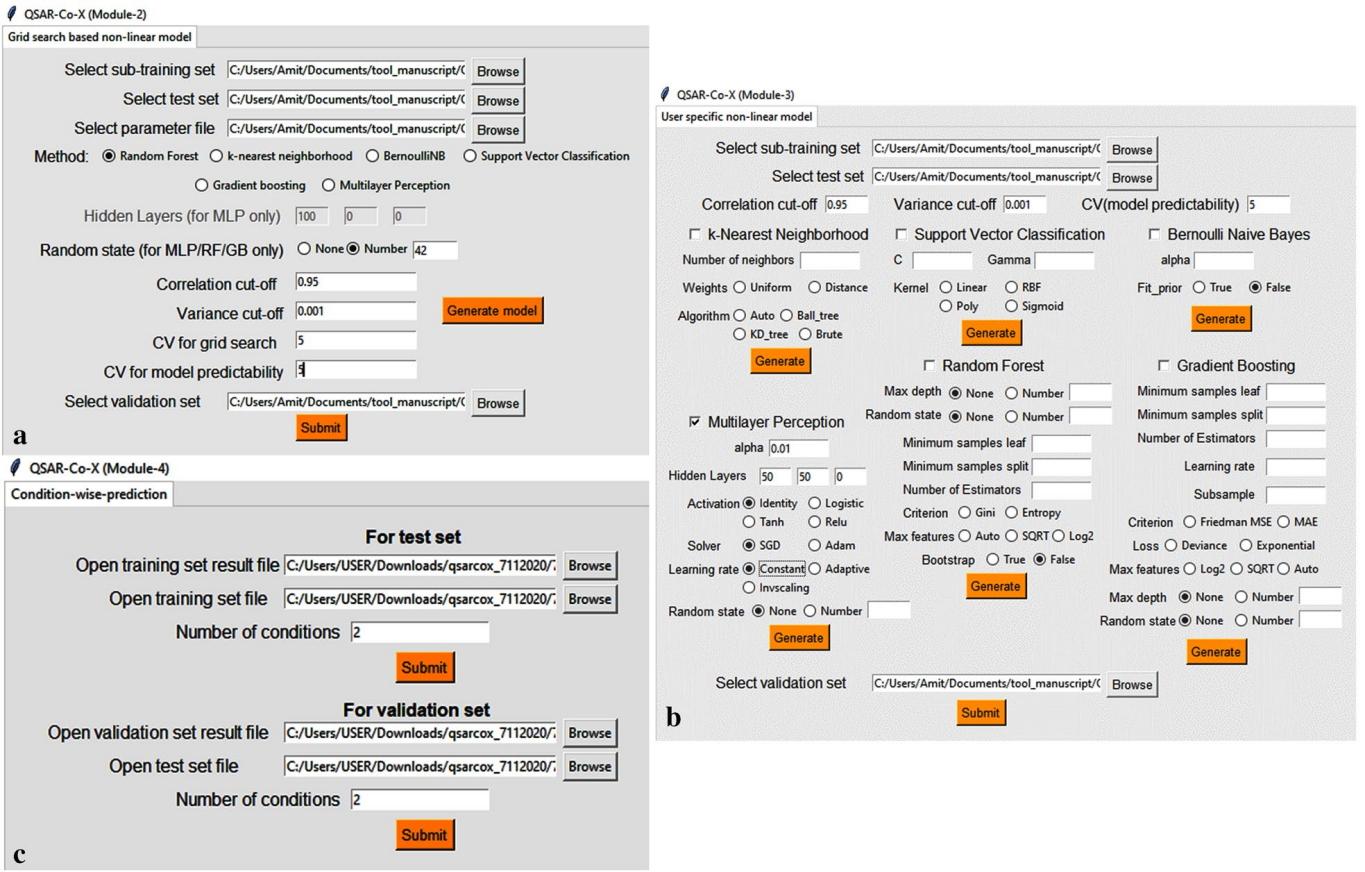
Screenshots of the Module 2 (**a**), Module 3 (**b**), and Module 4 (**c**) graphic user interface from toolkit QSAR-Co-X

Module 2 assists in setting up non-linear models using a grid search based hyperparameter optimisation scheme (see Fig. 2). Six machine learning tools have been so far implemented in *QSAR-Co-X*, namely: (a) *k*-Nearest Neighbourhood (*k*NN) [38], (b) Bernoulli Naïve Bayes (NB) classifier [39], (c) Support Vector Classifier (SVC) [40], (d) Random Forests (RF) [18], (e) Gradient Boosting (GB) [41], and f) Multilayer Perceptron (MLP) neural networks [42]. For all these non-linear modelling techniques, the Scikit-learn machine learning package is used [30, 31]. Similarly, the data pre-treatment option may be utilised in this module as well as in Module 3. In both these modules, the sub-training, test and validation sets set up with Module 1 of *QSAR-Co-X* are required to be uploaded one after another for development of the non-linear models.

In Module 2, a range of parameters of the machines learning tools are varied to obtain the most robust and predictive non-linear models, based on a *n*-fold (*i.e.,* user specific) cross-validation scheme using the *GridSearchCV* of Scikit-learn [30, 31]. In this module, a parameter file should be provided as.csv file that includes the parameter names with their values that are required to be optimised. In https://github.com/ncordeirfcup/QSAR-Co-X however, six such parameter files related to the various machine learning techniques are available, namely: grid_knn.csv, grid_nb.csv, grid_svc.csv, grid_mlp.csv, grid_rf.csv and grid_gb.csv. The parameter names and their values mentioned in these files are shown in Table 2 below. The files were prepared based upon the importance of the parameters as well as considering our previous experience regarding overall time requirements for the calculations. Nevertheless, the scope of this module is not only limited to these parameters (and values), because the users may select their own options for hyperparameter tuning by simply altering them. After selecting the best parameters, internal validation of the sub-training set is carried out by *n*-fold (*i.e.*, user-specific) cross validation, as well as external validation of both the test and validation sets. Similar to Module 1, the statistical results obtained for the non-linear models are automatically generated along with the optimised parameters, as well as ROC curves for the test and validation sets. Similar to *QSAR-Co*, the non-linear models’ AD is determined by the confidence estimation approach [43, 44].

**Table 2:** Hyper-parameters tuning options available in QSAR-Co-X toolkit

Technique	Parameters tuning^a^
RF	Bootstrap: True/ False^b^
	Criterion: Gini, Entropy,
	Maximum depth: 10, 30, 50, 70, 90, 100, 200, None
	Maximum features: Auto, Sqrt
	Minimum samples leaf: 1, 2, 4
	Minimum samples split: 2, 5, 10
	Number of estimators: 50, 100, 200,500
*k*NN	Number of neighbours: 1–50
	Weight options: Uniform, Distance
	Algorithms: Auto, Ball tree, kd_tree, brute
Bernoulli NB	Alpha:1, 0.5, 0.1
	Fit_prior: True, False
SVC	C: 0.1, 1, 10, 100, 1000
	Gamma: 1, 0.1, 0.01, 0.001
	Kernel: RBF, Linear, Poly, Sigmoid
MLP	Hidden layer sizes: To be specified by the user
	Activation: Identity, Logistic, Tanh, Relu
	Solver: SGD, Adam
	Alpha: 0.0001, 0.001, 0.01, 1
	Learning rate: Constant, Adaptive, Invscaling
GB	Loss: deviance, exponential
	Learning rate: 0.01, 0.05, 0.1, 0.2
	Min samples split: 0.1,0.2,0.3,0.4,0.5
	Minimum samples leaf: 0.1,0.2,0.3,0.4,0.5
	Maximum depth: 3,5,8
	Maximum features: Log2, Sqrt
	Criterion: Friedman MSE, MAE
	Subsample: 0.5, 0.6, 0.8
	Number of estimators: 50,100,200,300

^a^For further details on these parameters, check the manual associated with the toolkit in https://github.com/ncordeirfcup/QSAR-Co-X

^b^This option is automatically selected

### Module 3 (NLU)−user specific parameter settings

The functionality of Module 3 (Fig. 2) is the same as that of Module 2, *i.e*., development of non-linear models. However, in Module 3, the user may specify the parameter settings. Since grid search is a time consuming but recommended technique, this module could be used for fast generation of the non-linear models. Even after hyper-parameter tuning, the optimised parameters obtained from Module 2 can be specified in Module 3 for rapid obtention of the optimised models. Other utilities of Module 3, such as calculation of statistics for internal and external validation, pre-treatment of data-files, and making ROC curves for both the test and the validation sets, are similar to Module 2.

### Module 4 (CWP)−condition-wise prediction

The *QSAR-Co-X* toolkit includes this automated and simple analysis tool that can be used for checking the mt-QSAR obtained results. Indeed, since the mt-QSAR modelling implemented in *QSAR-Co-X* leads to a unique model for datasets containing several experimental and/or theoretical conditions, one may need to assess how much the derived model is predictive to a specific condition. Module 4 (see Fig. 2) is then to be employed to inspect the models’ performance against each condition, due to different reasons. For example, if the user often ends up with almost equally predictive models, he/she might select one of them on the basis of being more predictive towards a particular condition of interest. Moreover, the conditions over which the model is less predictive may be removed to obtain more predictive and/or more significant models. Finally, experimental or theoretical conditions with negligible number of cases may in addition be identified through this analysis and if the derived model is found less predictive towards such conditions, these may be removed also to rebuild the model.

**Fig. 3:**
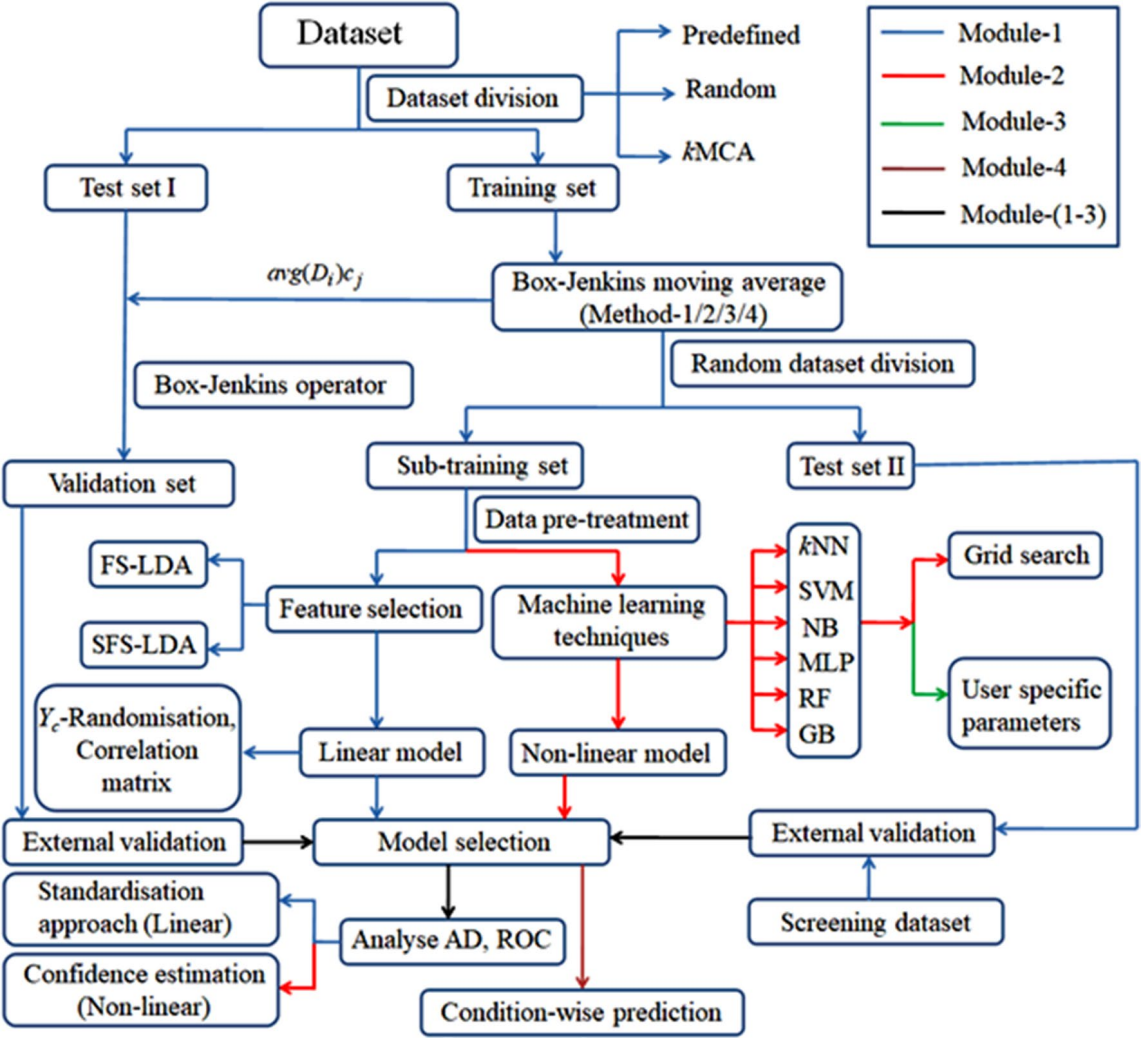
Illustration of the overall functionalities of toolkit QSAR-Co-X

The overall workflow of this new toolkit along with whole of its described modules can be seen in Fig. 3.

## Results

To check as well as to demonstrate the utilities of the developed *QSAR-Co-X* toolkit, four case studies pertaining to previously compiled datasets [9, 11, 26, 27] are examined in this section. For all of them, both the activity cut-off values and the descriptors employed in the original publications were used here (exact details about those can be found in the original papers). The main purpose of these four chosen case studies are as follows:

- Case study 1: Demonstrate how linear and non-linear mt-QSAR models may be developed with this toolkit.
- Case study 2: Show how different models may be generated using different data-splitting facilities of the toolkit.
- Case study 3: Describe how models may be generated using the various available Box-Jenkins operators.
- Case study 4: Perform a comparative analysis between the model development techniques of the former QSAR-Co and the new QSAR-Co-X toolkit.

### Case study-1 (CS1)

The first dataset comprises 726 inhibitors of four I phosphoinositide 3-kinase (PI3K) enzyme isoforms (PI3K*-α, -β, -γ, -δ*), the activities of which have been assayed against 34 mutated or wild human cell lines [11]. The experimental conditions considered in this dataset can be expressed as an ontology of the form *c*_*j*_ → (*bt*, *cl*, *mt*), *i.e.,* corresponding to the combination of the three following elements: *b*_*t*_ (biological enzyme target), *c*_*l*_ (cell line) and *m*_*t*_ (mutated or wild cell lines).

Compounds with IC_50_/K_i_ /K_d_ values ≤ 600 nM were assigned as active whereas the remaining data samples considered as inactive. The dataset contained 536 active (+ 1) and 190 (− 1) inactive compounds and the mt-QSAR models were developed for predicting the activity of inhibitor compounds against these four isoforms of PI3K under various experimental conditions.

#### Linear interpretable models

The dataset was first divided into a training and validation set using a random division scheme (22% of the data taken as the validation set, seed value = 2). Subsequently, the Box-Jenkins operator (Method1, Eq. 1) was applied to produce a sub-training set (*n*_*str*_ = 452), a test set (*n*_*ts*_ = 114) and a validation set (*n*_*vd*_ = 160), using a seed value of 2. The FS-LDA model was then derived with the following options: (a) correlation cut-off of 0.999, (b) variance cut-off of 0.001, (c) *p*-value to enter of 0.05, and (d) *p*-value to remove of 0.05. Meanwhile, the SFS-LDA model was built using the following: (a) correlation cut-off of 0.999, (b) variance cut-off of 0.001, (c) Floating = True, and (d) Scoring = Accuracy. For both models, a maximum of ten descriptors were allowed, the sub-training results of being shown in Supplementary Information (Additional file 1: Table S1). As can be seen in Table S1, the FS-LDA model shows a higher goodness-of-fit than the SFS-LDA model.

The FS-LDA model that was developed in the first attempt depicted high inter-collinearity with a maximum Pearson correlation coefficient (*r*) of 0.926 between two of its descriptors. Therefore, the maximum allowed paired-correlation coefficient was reduced to 0.90, and the final rebuilt model yielded a Wilk’s *λ* of 0.261. Similarly, the first SFS-LDA model developed also presented a high inter-collinearity between two of its descriptors (*r* > 0.98). Therefore, the later model was rebuilt by reducing the correlation cut-off to 0.95, and this revised SFS-LDA model depicted a much satisfactory inter-collinearity among descriptors (maximum *r* = 0.808). The overall predictivity of the linear models is depicted in Table 3.

**Table 3:** Overall predictivity of the linear models produced for CS1

Classification^a^	FS-LDA			SFS-LDA		
	Str^d^	Ts^e^	Vd^f^	Str^d^	Ts^e^	Vd^f^
TP	332	77	110	333	77	110
TN	102	32	36	106	33	36
FP	9	3	8	5	2	8
FN	9	2	6	8	2	6
Sn (%)	91.89	91.43	81.82	95.49	94.29	81.82
Sp (%)	97.36	97.47	94.83	97.65	97.47	94.83
Acc (%)	96.02	95.61	91.25	**97.12**	**96.49**	**91.25**
F1 score (%)	97.36	96.85	94.02	98.08	97.47	94.02
MCC^b^	0.892	0.896	0.778	0.923	0.917	0.778
AUROC^c^	0.946	0.944	0.883	0.966	0.959	0.883

The most significant results are highlighted in bold

^a^TP: True positive, TN: True negative, FP: False positive, FN: False negative, Sn: Sensitivity, Sp: Specificity, Acc: Accuracy.

^b^Matthews correlation coefficient.

^c^Score for the area under the receiver operating characteristic curve.

^d^Sub-training set.

^e^Test set.

^f^Validation set

As can be seen, the SFS-LDA model was found to be more predictive than the FS-LDA model. The average accuracy and MCC values found for the newly developed SFS-LDA model are 94.95% and 0.873, respectively. After analysing the AD computed by the standardisation approach, in the FS-LDA model, 15 data-points of the sub-training set, 6 data-points of the test set, and 5 data-points of the validation set are found to be outliers. While, in the SFS-LDA model, 43 sub-training set, 13 test set and 14 validation set samples emerged as structural outliers. Therefore, based on the results of AD, it may be inferred that the FS-LDA model was developed with descriptors that yield a considerably smaller number of structural outliers compared to the SFS-LDA model. The ROC plots of FS-LDA and SFS-LDA models generated with the current toolkit can be found in Supplementary Information (Additional file 1: Figure S1).

#### Non-linear models

This dataset was then subjected to non-linear model development using the *QSAR-Co-X* toolkit. For such a purpose, the hyperparameter tuning implemented in its Module 2 was employed. Details about the corresponding optimised parameters along with the accuracy values obtained for the sub-training, test and validation sets can be found in Supplementary Information (Additional file 1: Table S2). It can be observed that, except for Bernoulli NB, all other machine learning tools are able to produce highly predictive mt-QSAR models. However, the RF and GB tools lead to the most significant non-linear mt-QSAR models, judging from their internal and external validation parameters (*i.e*., accuracy in this case; see Table 4). Although the same accuracy is obtained for the validation set, on the basis of overall predictivity, the RF model is found to be slightly superior to the GB model. Table 4 shows the overall statistical predictivity of the latter two models, whereas the ROC plots for the validation and test sets are depicted in Supplementary Information (Additional file 1: Figure S2). Interestingly, the external predictivity of the RF model matches exactly with the FS-LDA model (*cf.* Table 3).

**Table 4:** Overall predictivity of the derived RF and GB models

Classification^a^	RF			GB		
	Str (fivefold CV) ^d^	Ts^e^	Vd^f^	Str (fivefold CV)^d^	Ts^e^	Vd^f^
TP	330	77	110	331	75	108
TN	98	32	36	97	32	38
FP	13	3	8	14	3	6
FN	11	2	6	10	4	8
Sn (%)	96.77	91.43	81.82	97.07	91.43	86.36
Sp (%)	88.29	97.47	94.83	87.39	94.94	93.10
Acc (%)	94.69	95.61	91.25	94.69	93.86	91.25
F1 score (%)	96.49	96.85	94.02	96.50	95.54	93.91
MCC^b^	–	0.896	0.778	–	0.857	0.784
AUROC^c^	–	0.944	0.883	–	0.932	0.897

^a^TP: True positive, TN: True negative, FP: False positive, FN: False negative, Sn: Sensitivity, Sp: Specificity, Acc: Accuracy

^b^Matthews correlation coefficient

^c^Score of area under the receiver operating characteristic curve

^d^Sub-training set

^e^Test set

^f^Validation set

Finally, Module 4 of *QSAR-Co-X* was applied for a condition-wise prediction of the FS-LDA model, and the obtained results are listed in Table 5. Note that a similar analysis might have been also performed with any of the non-linear models. Here, it should be mentioned that the present dataset pertains to as many as 34 experimental condition elements, and from Table 5 it can be observed that not all the latter appear in both the test and validation sets. However, owing to the high external predictivity of the model, most of these experimental elements are predicted with high accuracy values. Nevertheless, it can be additionally seen that samples pertaining to elements 18 and 24 are not only present in less number but are also poorly predicted. These samples may then be removed, or alternate models been generated with other techniques in which the predictivities for these experimental condition elements are higher. Similarly, a ‘condition-wise prediction’ analysis might also be performed using the derived non-linear models with the help of the present module. The results, *i.e.*, the output files generated for the FS-LDA, SFS-LDA, RF and GB models of CS1 are given in Additional file 2.

**Table 5:** Condition-wise prediction for the FS-LDA model built in CS1

SN	Experimental condition element (*c*_*j*_)			Test set		Validation set	
	*c*_*l*_	*m*_*t*_	*b*_*t*_	#Instances	%Accuracy	#Instances	%Accuracy
1	Normal- MCF7-neo/Her2	Non-mutant	PI3K-α	2	100	3	100
2	Normal-B-cells	Non-mutant	PI3K-δ	8	100	12	100
3	Normal-BT20	Non-mutant	PI3K-α	2	100	1	100
4	Normal-BT474	Mutant	PI3K-α	7	85.71	10	90
5	Normal-BT474	Non-mutant	PI3K-α	14	85.71	13	84.62
6	Normal-HCC1954	Non-mutant	PI3K-β	1	100	1	100
7	Normal-HCT116	Mutant	PI3K-α	4	100	2	100
8	Normal-HCT116	Non-mutant	PI3K-α	1	100	3	100
9	Normal-HEK293	Non-mutant	PI3K-β	1	100	na	na
10	Normal-HL60	Non-mutant	PI3K-α	3	66.67	6	100
11	Normal-HL60	Non-mutant	PI3K-β	5	100	2	50
12	Normal-HL60	Non-mutant	PI3K-γ	2	100	na	na
13	Normal-HL60	Non-mutant	PI3K-δ	na	na	6	83.33
14	Normal-HL60	Non-mutant	PI3K-γ	na	na	6	100
15	Normal-JeKo1	Non-mutant	PI3K-δ	4	100	4	100
16	Normal-MDA-MB-453	Mutant	PI3K-α	4	100	5	100
17	Normal-MDA-MB-468	Non-mutant	PI3K-β	3	100	10	100
**18**	**Normal-PBMC**	**Non-mutant**	**PI3K-δ**	**na**	**na**	**1**	**0**
19	Normal-PC3	Non-mutant	PI3K-α	7	100	12	91.67
20	Normal-PC3	Non-mutant	PI3K-β	2	100	na	na
21	Normal-PC3	Non-mutant	PI3K-γ	1	100	na	na
22	Normal-Ramos	Non-mutant	PI3K-δ	1	100	na	na
23	Normal-Ri-1	Non-mutant	PI3K-δ	na	na	5	80
**24**	**Normal-THP1**	**Non-mutant**	**PI3K-β**	**1**	**0**	**na**	**na**
25	Normal-THP1	Non-mutant	PI3K-δ	3	100	6	66.67
26	Normal-THP1	Non-mutant	PI3K-γ	1	100	na	na
27	Normal-U2OS	Non-mutant	PI3K-α	2	100	3	100
28	Normal-U87MG	Non-mutant	PI3K-α	7	100	15	86.67
29	Normal-U937	Non-mutant	PI3K-δ	1	100	na	na
30	PTEN-deficient-MDA-MB-468	Non-mutant	PI3K-β	5	100	7	100
31	PTEN-deficient-PC3	Non-mutant	PI3K-β	10	100	19	89.47
32	PTEN-deficient-U87MG	Non-mutant	PI3K-α	3	100	na	na
33	PTEN-Null-MDA-MB-468	Non-mutant	PI3K-β	8	100	8	100
34	PTEN-Null-PC3	Non-mutant	PI3K-α	1	100	na	na

The experimental condition elements not well predicted by the model are highlighted in bold

### Case study-2 (CS2)

The second case study aims at investigating the impact of data-distribution during the development of mt-QSAR models. Further, the significance of *Y*_*c*_ randomization as an extra criterion for justifying the robustness of linear models is aimed to be demonstrated also. A previously collected dataset [26] will be employed, which contains 46,229 datapoints describing the anti-bacterial activity against Gram-negative pathogens and in vitro safety profiles related to absorption, distribution, metabolism, elimination, and toxicity (ADMET) properties. This dataset pertains to four experimental condition elements (*c*_*j*_), namely: *b*_*t*_ (biological target), *m*_*e*_ (measure of effect), *a*_*i*_ (assay information), and *t*_*m*_ (target mapping). Additionally, each datapoint includes a probabilistic factor *p*_*c*_ to account for the degree of reliability of the experimental information. Each case in the data set was assigned as one out of two possible categories, namely positive (+ 1) or negative (− 1). Cut-off values for different measures of toxicity effects of compounds are provided in Supplementary Information (Additional file 1: Table S4).

Two different models were generated and in the first case the probabilistic factor *p*_*c*_ was discarded, and the models developed using ‘Method1’. Then, in the second case, the models were developed considering the influence of *p*_*c*_ and due to its presence, the Box-Jenkins operator based on ‘Method4’ (Eq. 6) was employed. For both cases, we applied three dataset distribution methods available in *QSAR-Co-X* for splitting the data into the training and validation sets. In the first method (*i.e.*, pre-defined sets), the training (75% of the data) and validation (25% of the data) sets coming from the original work were used. In the second method (*i.e.*, random division), 25% of the data was placed in the validation set using a random seed value of 2. In the third method (*i.e.*, *k*MCA based division), the data was divided into ten clusters and, from each of these, 25% of the data was selected as the validation set, and subsequently each training set was divided into sub-training (80%) and test (20%) sets using a random seed value of 3. For each of these data distributions, SFS-LDA models were developed using the current toolkit with the following parameters: (a) correlation cut-off of 1.0, (b) variance cut-off of 0.001, (c) maximum steps = 6, (d) Floating = True, and (e) Scoring = Accuracy. The statistical results then gathered as well as the ROC plots for the derived three linear models can be found in Supplementary Information (Additional file 1: Figure S3, Tables S3 and S4). The latter plots along with the corresponding AUROC values allows one to infer the classification ability of the generated mt-QSAR models.

As one may observe from Additional file 1: Table S4, irrespectively of the data-distribution method used, the models generated with ‘Method4’ display slightly better statistical parameters. That thus suggests that the probabilistic factor considered in the original investigation truly influences in determining the response variable.

Focusing now only on ‘Method4’ based models, the Wilk’s *λ* values obtained for these pre-defined, random and *k*MCA division-based models were 0.438, 0.432 and 0.440, respectively. Such low values for the sub-training sets show that all these models display an adequate discriminatory power and a satisfactory goodness-of-fit. In addition, at first sight (Additional file 1: Table S4), there are no significant differences between these models as regards their statistical quality indicating that no matter which data-distribution method is considered, the quality of the linear model remains almost similar. However, after verifying the internal and external validation results, the random division-based model is seen to be the best linear mt-QSAR model. Further, the degree of collinearity among the variables of the model is not too high, the maximum correlation coefficient between two of its descriptors being 0.831. To further judge the statistical significance of this model, we applied the *Y*_*c*_ randomization scheme implemented in *QSAR-Co-X*. To do so, the response variable and experimental elements were randomised 100 times, and the resulting 100 randomised data matrices were then subjected to the same Box-Jenkins operator (*i.e.*, ‘Method4’) used for generating the original model. Subsequently, 100 models were created with the randomised sub-training set using the descriptors of the original model. The average Wilk’s *λ* (*λ*_*r*_) and average accuracy (*Accuracy*_*r*_) found for such models were 0.999 and 58.09, respectively, which compared to those attained for the original model (*i.e.*, 0.432 and 96.37) confirm that the latter is unique and lacks chance correlations. The results, *i.e.*, the output files from the current toolkit, of these SFS-LDA models for CS2 are shown in Additional file 3.

### Case study-3 (CS3)

The purpose of third case study is to disclose how different Box-Jenkins’s operators may have an impact on the statistical quality of the derived models. The dataset of CS3 was retrieved from a recently published work in which the toxicity of 260 pesticides have been targeted by mt-QSAR modelling with artificial neural networks (ANN) [27]. The dataset comprised a total of 3610 datapoints related to four *primary* experimental condition elements (*c*_*j*_), namely: *m*_*e*_ (measure of toxicity), *b*_*s*_ (bioindicator species), *a*_*g*_ (assay guideline) and *e*_*p*_ (exposure period). For detailed information about the cut-off values employed for the different measures of toxicity effects, please refer to the Supplementary Information (Additional file 1: Table S5). Further details about *m*_*e*_, *b*_*s*_, *a*_*g*_ and *e*_*p*_ can be obtained from the original work [27]. The dataset contained 1992 toxic (+ 1) and 1618 nontoxic (− 1) compounds. Additionally, three other experimental condition elements have been taken into consideration while modelling, these being the concentration lethality (*l*_*c*_), target mapping (*t*_*m*_) and time classification (*t*_*c*_). The latter three may be specified as *secondary* experimental elements ($c_{{j_{2} }}$) due simply to the fact that *l*_*c*_, *t*_*m*_ and *t*_*c*_ are related to *m*_*e*_, *b*_*s*_ and *e*_*p*_, respectively. On the basis of these related primary and secondary experimental elements, three probabilistic factors were calculated in that work as follows [27]:

8 $$p{(}m_{e} {)}_{{l_{c} }} = \frac{{n_{{\text{T}}} {(}m_{e} {)}}}{{N_{{\text{T}}} {(}l_{c} {)}}}$$

9 $$p{(}b_{s} {)}_{{t_{m} }} = \frac{{n_{{\text{T}}} {(}b_{s} {)}}}{{N_{{\text{T}}} {(}t_{m} {)}}}$$

10 $$p{(}e_{p} {)}_{{t_{c} }} = \frac{{n_{{\text{T}}} {(}e_{p} {)}}}{{N_{{\text{T}}} {(}t_{c} {)}}}$$

where $n_{{\text{T}}} {(}c_{j} {)}$ and $N_{{\text{T}}} {(}c_{{j_{2} }} {)}$ stand for the number of the training set samples, including toxic and non-toxic data points, within the primary and secondary experimental elements, respectively.

In that work, another probabilistic factor was also included based on the following equation [27]:

11 $$p{(}a_{g} {)} = \frac{{n{(}a_{g} {)}}}{{N_{{\text{T}}} }}$$

where *N*_T_ stands for the total number of samples in the training set, and notably this equation is just like Eq. 5, already implemented within one of the Box-Jenkins operators (‘Method3’) in *QSAR-Co-X*, because it merely corresponds to a normalisation by all the number of elements.

Each of these probabilistic factors may be simply denoted as $p{(}c_{j} {)}$ and so, the final deviation descriptors employed in such a work [27] are similar to the standardised modified descriptors presented in Eq. 4. Yet these final descriptors embody a more complex moving average operator that is not implemented in *QSAR-Co-*X (*cf*. Equations 3–6). Yet ‘Method4’ (Eq. 6) may still be applied with a slight modification to obtain the same modified descriptors used in that work. To that end, the python code of ‘Method4’ was adapted to calculate the modified descriptors (‘Method4 modified’, *cf.* Table 6) from the starting descriptors reported in such work [27]. Then, non-linear mt-QSAR models were developed using a pre-defined data-distribution, *i.e.* to use the same training and validation sets employed in the original work [27]. Eighty percent of the training dataset was treated as the sub-training set whereas the remaining was used as the test set for setting up RF based non-linear models. However, instead of employing pre-selected features for developing the non-linear models, just as it has been done on that original work, here we resort to a maximum descriptor space for model generation. In order to remove less descriptive highly correlated features, a data pre-treatment was carried out by setting the correlation cut-off in 0.95 and the variance cut-off in 0.001. In addition, a fivefold cross-validation was used for grid search as well as for inspecting the internal predictivity of the sub-training set. After developing the model using the adapted ‘Method4’, this model was also compared to models derived based on other operators (*i.e*., with the original Methods1–4) implemented in *QSAR-Co-X*. However, to calculate the descriptors using Methods 1–3, the probabilistic factors (*i.e.*, the original $p{(}m_{e} {)}_{{l_{c} }}$, $p{(}b_{s} {)}_{{t_{m} }}$, and $p{(}e_{p} {)}_{{t_{c} }}$ factors) could not be accommodated. Therefore, for these methods the influence of all secondary experimental elements was discarded. However, these probabilistic factors were considered in the model developed by Method4. The results of the RF models developed with all five type of moving average operators and related deviation descriptors are shown in Table 6.

**Table 6:** Overall performance of the final RF models in CS3

Classification^b^	Method4 modified			Method1 (Eq. 1) ^d^			Method2 (Eq. 3) ^d^			Method3 (Eq. 4) ^d^				Method4 (Eq. 6)		
	Str^e^	Ts^f^	Vd^g^	Str^e^	Ts^f^	Vd^g^	Str^e^	Ts^f^	Vd^g^	Str^e^	Ts^f^	Vd^g^	Str^e^		Ts^f^	Vd^g^
TP	1011	252	419	1023	251	420	1015	250	412	1030	243	418	1020		249	427
TN	744	190	303	753	193	315	746	193	313	730	187	303	731		188	302
FP	230	56	95	221	53	83	228	53	85	244	59	95	243		58	96
FN	191	46	73	179	47	72	187	48	80	172	55	74	182		49	65
Sn (%)	84.11	77.24	76.13	85.11	78.45	79.15	84.44	78.45	78.64	85.69	76.02	76.13	84.86		76.42	75.88
Sp (%)	76.39	84.56	85.16	77.31	84.22	85.37	76.59	83.89	83.74	74.95	81.54	84.96	75.05		83.56	86.79
Acc (%)	80.65	81.25	81.12	**81.62**	**81.62**	**82.58**	80.93	81.43	81.46	80.88	79.04	81.01	80.47		80.33	81.91
F1 score (%)	82.77	83.17	83.3	83.65	83.39	84.42	83.03	83.19	83.32	83.2	81	83.18	82.76		82.31	84.13
MCC^**c**^	0.608	0.621	0.617	**0.627**	**0.628**	**0.647**	0.613	0.625	0.625	0.612	0.576	0.614	0.604		0.602	0.633

^a^The most significant results are highlighted in bold. All the models were generated using random state ‘None’ in Module 2 of the toolkit

^b^TP: True positive, TN: True negative, FP: False positive, FN: False negative, Sn: Sensitivity, Sp: Specificity, Acc: Accuracy

^c^Matthews correlation coefficient

^d^No secondary experimental elements used

^e^Sub-training set

^f^Test set

^g^Validation set

As seen, the models obtained here reveal to display more predictive ability than that of the model reported in the original investigation (MCC score of 0.524 for the test set) [27]. Nevertheless, the latter is more interpretable since only a limited number of features was used for its development. Therefore, a direct comparison of the reported model with the current RF models is not feasible, yet nor it is the purpose of the current case study. Rather, our aim here is to disclose the importance of different operators implemented in *QSAR-Co-X*. Even though the variations in the operators did not have significant impact on the statistical quality of all these models, the mt-QSAR model obtained from ‘Method1’ is found to produce the best solution relying on both internal and external predictivity. However, this outcome is based only on one data-distribution technique and one machine learning method. Therefore, no final conclusion might be drawn regarding the utility of these operators. The case study however demonstrates that the multiple operators implemented in *QSAR-Co-X* may be utilised to judge which option is most suitable for a specific data. The results, *i.e.*, the output files from the current toolkit, obtained from RF model by applying Method1 for CS3 are given in Additional file 4.

Finally, it is important to remark here that, the previously reported model was developed by resorting to a commercial software.

### Case study-4 (CS4)

Case studies 1–3 were examined mainly to demonstrate some of the basic utilities of QSAR-Co-X. In the final case study, we attempted however to compare the performances of previously reported QSAR-Co models with newly created QSAR-Co-X models. For such purpose, we collected a previously reported dataset containing 2,123 peptides (amino acid length 4–119) with antibacterial activities against multiple Gram-negative bacterial strains and cytotoxicity against multiple cell types [9]. This dataset pertains to two experimental condition elements (*c*_*j*_), namely: *b*_*s*_ (biological target) and *m*_*e*_ (measure of effect). Each peptide in the data set was assigned to one out of two possible categories, namely: positive (+ 1) − *i.e.*, indicating high antibacterial activity or low cytotoxicity, or negative (− 1) − *i.e.*, showing low antibacterial activity or high cytotoxicity. The cut-off values to annotate a peptide as positive were: MIC ≤ 14.97 μM, or CC50 ≥ 60.91 μM, or HC50 ≥ 105.7 μM. For more details, please refer to the original investigation [9]. Mt-QSAR modelling of this dataset has already been performed using the QSAR-Co tool [15], being the linear model developed with the GA-LDA technique and the non-linear model with the RF technique. In this case study, three additional linear models were built using QSAR-Co-X, keeping the same maximum number of descriptors (*i.e*., four) and data-distributions. Table 7 shows the statistical parameters obtained for all these models. Note that two LDA models were set up by applying SFS for feature selection with the two different scoring parameters (*i.e.*, Accuracy and AUROC).

**Table 7:** Overall performance of the final linear models for CS4

Classification^b^	QSAR-Co^a^			QSAR-Co-X								
	GA-LDA			FS-LDA			SFS-LDA			SFS-LDA		
							(Scoring: Accuracy)			(Scoring: AUROC)		
	Str^c^	Ts^d^	Vd^e^	Str^c^	Ts^d^	Vd^e^	Str^c^	Ts^d^	Vd^e^	Str^c^	Ts^d^	Vd^e^
TP	941	418	315	940	422	323	934	413	322	930	407	328
TN	932	389	311	925	388	302	947	393	309	956	406	317
FP	67	33	16	74	34	25	52	29	18	43	16	10
FN	97	33	40	98	29	32	104	38	33	108	44	27
Sn (%)	90.65	92.68	88.73	92.59	91.94	92.35	94.79	93.13	94.49	95.7	96.21	96.94
Sp (%)	93.29	92.18	95.11	90.56	93.57	90.99	89.98	91.57	90.7	89.59	90.24	92.39
Acc (%)	91.95	92.44	91.79	91.56	92.78	91.64	92.34	92.32	92.52	**92.59**	**93.13**	**94.57**
MCC^f^	0.839	0.849	0.838	0.831	0.855	0.833	0.848	0.847	0.851	0.853	0.864	0.893

The most significant results are highlighted in bold

^a^Model previously reported in [21]

^b^TP: True positive, TN: True negative, FP: False positive, FN: False negative, Sn: Sensitivity, Sp: Specificity, Acc: Accuracy

^c^Sub-training set

^d^Test set

^e^Validation set

^f^Matthews correlation coefficient

The Wilks’ lambda (λ) value obtained for the original developed GA-LDA model is 0.422, whereas those of the FS-LDA, SFS-LDA (Scoring: Accuracy) and SFS-LDA (Scoring: AUROC) models are 0.421, 0.444 and 0.451, respectively. As seen in Table 7, among the QSAR-Co-X linear models, the SFS-LDA model generated with the AUROC scoring parameter is found to be the best one, judging from its overall predictivity results. Furthermore, overall predictivity of this model is significantly higher than that of the GA-LDA model previously reported [15].

Similarly, in this case study, we also developed two non-linear models through the RF and GB techniques. It is important to mention here that QSAR-Co does not provide any option for hyperparameter optimisation and therefore the earlier reported RF model has been generated without it. On the other hand, the models generated by QSAR-Co-X were set up with hyperparameter optimisation by supplying the values for the parameter settings in its Module 2. Table 8 shows the attained results for these models.

**Table 8:** Overall performance of the final non-linear models for case study 4

Classification ^b^	RF (without HPO^c^/QSAR-Co)^d^			RF (with HPO/QSAR-Co-X)			GB (with HPO/QSAR-Co-X)		
	Str(tenfold CV)^e^	Ts^f^	Vd^g^	Str(tenfold CV)^e^	Ts^f^	Vd ^g^	Str (tenfold CV)^e^	Ts^f^	Vd^g^
TP	994	431	341	969	433	343	996	443	346
TN	953	405	317	936	405	316	949	406	318
FP	46	17	10	63	17	11	50	16	9
FN	44	20	14	69	18	12	42	8	9
Sn (%)	95.76	95.57	96.06	93.35	95.97	96.64	95.95	96.21	97.46
Sp (%)	95.4	95.97	96.94	93.69	96.01	96.62	94.99	98.22	97.25
Acc (%)	95.58	91.52	96.48	93.52	95.99	96.63	**95.48**	**97.25**	**97.36**
MCC^h^	0.912	0.915	0.93	0.884	0.920	0.932	0.91	0.945	0.947

^a^The most significant results are highlighted in bold. QSAR-Co-X were generated using random state 1 in Module 2 of the toolkit

^b^TP: True positive, TN: True negative, FP: False positive, FN: False negative, Sn: Sensitivity, Sp: Specificity, Acc: Accuracy

^c^HPO: Hyperparameter optimisation

^d^Model previously reported in [15]

^e^Sub-training set

^f^Test set

^g^Validation set

^h^Matthews correlation coefficient

By inspecting the statistical parameters given in Table 8, it is clear that the GB model affords the best predictivity and leads to a significant improvement in the external predictive accuracy when compared to that of the previously reported RF model generated with QSAR-Co. However, it is noteworthy that the significance of this GB based model is not only limited to its better performance. Since this model has been developed with hyperparameter optimization, its overall acceptability is much higher than the RF model generated with QSAR-Co, without any tuning of hyperparameters [45, 46]. On the whole, the results shown in Tables 7, 8 clearly suggest that the QSAR-Co-X toolkit provides some very useful strategies for setting up linear and non-linear mt-QSAR models.

The results of the SFS-LDA and GB models, *i.e.*, the output files from the current toolkit, obtained for CS4 are given in the Supplementary Information (Additional file 5).

## Conclusions

In this work, we described the user-friendly open-source *QSAR-Co-X* toolkit that is an extension of our previously launched java-based tool *QSAR-Co* [15], and has a number of advantages over the latter to support mt-QSAR modelling efforts. Indeed, the current toolkit move a step forward by including more updated and advanced strategies, namely in what concerns data-distribution options, schemes for calculation of modified descriptors, feature selection algorithms, machine learning methods, validation strategies as well as analysis techniques. The *QSAR-Co-X* toolkit is based on Python, which is undoubtedly one of the most popular and highly accessed programming languages, especially in the field of data science [22]. The current toolkit utilises some well-known Python based libraries, such as NumPy [47], SciPy [48], Pandas [49], Matplotlib [50], Tkinter (https://anzeljg.github.io/rin2/book2/2405/docs/tkinter/index.html), and Scikit-learn [30, 31]. The codes of the toolkit are made available in public domain so that, necessary modifications/updates may be easily implemented during their utilisation. Similar to *QSAR-Co*, this toolkit relies primarily on Box-Jenkins based mt-QSAR modelling, which has been proved to be highly efficient in handling large datasets pertaining to various experimental and/or theoretical conditions[10–15, 20, 26–28, 51]. Further, the ability to explore all of its code tools simultaneously, as well as the graphical user interface itself, provide simple and efficient solutions to the main practical challenges implicated in mt-QSAR modelling. The latter was clearly shown by testing its functionalities on four case studies. Indeed, we were able to demonstrate the basic utilities of its tools and at the same time, depicted also how different feature selection algorithms, machine learning methods, dataset division options and different Box-Jenkins’s operators may play crucial roles in the development of more predictive mt-QSAR models. The toolkit allows the users to save the developed models and use these for predicting properties of new external chemicals. Clearly, future investigations using various datasets will lead to a better understanding about the utilities and short-comings of the functionalities of the present toolkit and will naturally give rise to its upgrading. Yet, on the whole, the toolkit presented here has the potential of becoming a widely used platform for easily setting up predictive mt-QSAR models.

## Supplementary Information

**Additional file 1. **File containing the QSAR-Co-X generated ROC plots (Figures S1–3) and additional information related to the several case studies (Tables S1–5).

**Additional file 2. **Folder (CS_1) containing the results (*i.e.*, the output files from the current toolkit) of the FS-LDA, SFS-LDA, RF and GB models for case study 1.

**Additional file 3. **Folder (CS_2) containing both the input files and the results (*i.e.*, the output files from the current toolkit) of the SFS-LDA models for Case study-2.

**Additional file 4. **Folder (CS_3) containing both the input files and the results (*i.e*., the output files from the current toolkit) obtained from the RF model by applying Method1 for Case study-3.

**Additional file 5. **Folder (CS_4) containing the input file of SFS-LDA and GB models and the results (*i.e*., the output files from the current toolkit) obtained from the SFS-LDA for Case study-4.

## Data Availability

Project name: QSAR-Co-X. Project home page: The source code of the toolkit along with its manual and reference data files are available from https://github.com/ncordeirfcup/QSAR-Co-X. Operating system(s): Platform independent. Programming language: Python. Other requirements: NumPy, SciPy, Pandas, Matplotlib, Tkinter and Scikit-learn. License: GNU GPL version 3. Any restrictions to use by non-academics: None.

## References

[1] Muratov EN, Bajorath J, Sheridan RP, Tetko IV, Filimonov D, Poroikov V, Oprea TI, Baskin II, Varnek A, Roitberg A, Isayev O, Curtalolo S, Fourches D, Cohen Y, Aspuru-Guzik A, Winkler DA, Agrafiotis D, Cherkasov A, Tropsha A (2020). QSAR without borders. Chem Soc Rev.

[2] Lewis RA, Wood D (2014). Modern 2D QSAR for drug discovery. WIRE-Comput Mol Sci.

[3] Neves BJ, Braga RC, Melo CC, Moreira JT, Muratov EN, Andrade CH (2018). QSAR-based virtual screening: advances and applications in drug discovery. Front Pharmacol.

[4] Gramatica P (2020). Principles of QSAR Modeling: Comments and suggestions from personal experience. Int J Quant Struc-Prop Relation.

[5] Toropov AA, Toropova AP (2020). QSPR/QSAR: State-of-art, weirdness, the future. Molecules.

[6] Polanski J, Roy K (2017). Big data in structure-property studies—from definitions to models. Advances in QSAR Modeling. Challenges and Advances in Computational Chemistry and Physics.

[7] Speck-Planche A (2018). Recent advances in fragment-based computational drug design: tackling simultaneous targets/biological effects. Future Med Chem.

[8] Speck-Planche A, Cordeiro MNDS (2017). Advanced in silico approaches for drug discovery: mining information from multiple biological and chemical data through mtkQSBER and pt-QSPR strategies. Curr Med Chem.

[9] Kleandrova VV, Ruso JM, Speck-Planche A, Cordeiro MNDS (2016). Enabling the discovery and virtual screening of potent and safe antimicrobial peptides. Simultaneous prediction of antibacterial activity and cytotoxicity. ACS Comb Sci.

[10] Halder AK, Natalia M, Cordeiro MNDS (2019). Probing the environmental toxicity of deep eutectic solvents and their components: An in silico modeling approach. ACS Sust Chem Eng.

[11] Halder AK, Cordeiro MNDS (2019). Development of multi-target chemometric models for the inhibition of class i pi3k enzyme isoforms: a case study using QSAR-Co tool. Int J Mol Sci.

[12] Speck-Planche A (2019). Multicellular target QSAR model for simultaneous prediction and design of anti-pancreatic cancer agents. ACS Omega.

[13] Speck-Planche A, Scotti MT (2019). BET bromodomain inhibitors: fragment-based in silico design using multi-target QSAR models. Mol Divers.

[14] Kleandrova VV, Scotti MT, Scotti L, Nayarisseri A, Speck-Planche A (2020). Cell-based multi-target QSAR model for design of virtual versatile inhibitors of liver cancer cell lines. SAR QSAR Environ Res.

[15] Ambure P, Halder AK, Diaz HG, Cordeiro MNDS (2019). QSAR-Co: An open source software for developing robust multitasking or multitarget classification-based QSAR models. J Chem Inf Model.

[16] Rogers D, Hopfinger AJ (1994). Application of genetic function approximation to quantitative structure-activity-relationships and quantitative structure-property relationships. J Chem Inf Comput Sci.

[17] Ambure P, Aher RB, Gajewicz A, Puzyn T, Roy K (2015). "NanoBRIDGES" software: Open access tools to perform QSAR and nano-QSAR modeling. Chemometrics Intellig Lab Syst.

[18] Breiman L (2001). Random forests. Mach Learn.

[19] Organization for Economic Co-Operation and Development (OECD). Guidance document on the validation of (quantitative) structure-activity relationship ((q)sar) models; OECD Series on Testing and Assessment 69; OECD Document ENV/JM/ MONO2007, pp 55−65.

[20] Halder AK, Giri AK, Cordeiro MNDS (2019). Multi-Target chemometric modelling, fragment analysis and virtual screening with erk inhibitors as potential anticancer agents. Molecules.

[21] Khan PM, Roy K (2018). Current approaches for choosing feature selection and learning algorithms in quantitative structure-activity relationships (QSAR). Expert Opin Drug Disc.

[22] Van Rossum G, Drake FL (2009). Python 3 Reference Manual.

[23] Gore PA, Tinsley HEA, Brown SD (2000). Cluster Analysis. Handbook of applied multivariate statistics and mathematical modeling.

[24] Mauri A, Consonni V, Pavan M, Todeschini R (2006). Dragon software: An easy approach to molecular descriptor calculations. MATCH Commun Math Comput Chem.

[25] Valdes-Martini JR, Marrero-Ponce Y, Garcia-Jacas CR, Martinez-Mayorga K, Barigye SJ, Almeida YSV, Perez-Gimenez F, Morell CA (2017). QuBiLS-MAS, open source multi-platform software for atom- and bond-based topological (2D) and chiral (2.5D) algebraic molecular descriptors computations. J Cheminform.

[26] Speck-Planche A, Cordeiro MNDS (2017). De novo computational design of compounds virtually displaying potent antibacterial activity and desirable in vitro ADMET profiles. Med Chem Res.

[27] Speck-Planche A, Roy K (2020). Multi-scale QSAR approach for simultaneous modeling of ecotoxic effects of pesticides. Ecotoxicological QSARs.

[28] Speck-Planche A (2018). Combining ensemble learning with a fragment-based topological approach to generate new molecular diversity in drug discovery: In silico design of Hsp90 inhibitors. ACS Omega.

[29] Menzies T, Kocagüneli E, Minku L, Peters F, Turhan B, Menzies T, Kocagüneli E, Minku L, Peters F, Turhan B (2015). Complexity: using assemblies of multiple models. Sharing data and models in software engineering.

[30] Hao JG, Ho TK (2019). Machine learning made easy: a review of scikit-learn package in python programming language. J Educ Behav Stat.

[31] Pedregosa F, Varoquaux G, Gramfort A, Michel V, Thirion B, Grisel O, Blondel M, Prettenhofer P, Weiss R, Dubourg V, Vanderplas J, Passos A, Cournapeau D, Brucher M, Perrot M, Duchesnay E (2011). Scikit-learn: Machine learning in python. J Mach Learn Res.

[32] Wilks SS (1932). Certain generalizations in the analysis of variance. Biometrika.

[33] Hans-Vaugn DL, Lomax RG (2020). An introduction to statistical concepts.

[34] Boughorbel S, Jarray F, El-Anbari M (2017). Optimal classifier for imbalanced data using Matthews Correlation Coefficient metric. PLoS ONE.

[35] Fawcett T (2006). An introduction to ROC analysis. Pattern Recognit Lett.

[36] Hanczar B, Hua JP, Sima C, Weinstein J, Bittner M, Dougherty ER (2010). Small-sample precision of ROC-related estimates. Bioinformatics.

[37] Roy K, Kar S, Ambure P (2015). On a simple approach for determining applicability domain of QSAR models. Chemometr Intell Lab Sys.

[38] Cover TM, Hart PE (1967). Nearest neighbor pattern classification. IEEE Trans Inf Theory.

[39] McCallum A, Nigam K (2001). A comparison of event models for naive bayes text classification. Work Learn Text Categ.

[40] Boser BE, Guyon IM, Vapnik VN A training algorithm for optimal margin classifiers. In Proceedings of the fifth annual workshop on Computational learning theory ACM 144–152.

[41] Friedman JH (2001). Greedy function approximation: a gradient boosting machine. Ann Stat.

[42] Huang GB, Babri HA (1998). Upper bounds on the number of hidden neurons in feedforward networks with arbitrary bounded nonlinear activation functions. IEEE Trans Neural Netw.

[43] Ambure P, Bhat J, Puzyn T, Roy K (2019). Identifying natural compounds as multi-target-directed ligands against Alzheimer's disease: an in silico approach. J Biomol Struct Dyn.

[44] Mathea M, Klingspohn W, Baumann K (2016). Chemoinformatic classification methods and their applicability domain. Mol Inform.

[45] Probst P, Boulesteix AL, Bischl B (2019). Tunability: importance of hyperparameters of machine learning algorithms. J Mach Learn Res.

[46] Wu J, Chen X-Y, Zhang H, Xiong L-D, Lei H, Deng S-H (2019). Hyperparameter optimization for machine learning models based on bayesian optimization. J Electr Sci Technol.

[47] van der Walt S, Colbert SC, Varoquaux G (2011). The NumPy array: a structure for efficient numerical computation. Comput Sci Eng.

[48] Virtanen P, Gommers R, Oliphant TE, Haberland M, Reddy T, Cournapeau D, Burovski E, Peterson P, Weckesser W, Bright J, van der Walt SJ, Brett M, Wilson J, Millman KJ, Mayorov N, Nelson ARJ, Jones E, Kern R, Larson E, Carey CJ, Polat I, Feng Y, Moore EW, VanderPlas J, Laxalde D, Perktold J, Cimrman R, Henriksen I, Quintero EA, Harris CR, Archibald AM, Ribeiro AH, Pedregosa F, van Mulbregt P, Contributors S (2020). SciPy 1.0: Fundamental algorithms for scientific computing in Python. Nat Methods.

[49] McKinney W (2010) Data structures for statistical computing in python, In: Proceedings of the 9th Python in Science Conference, Austin, Texas, 28 June-3 July 2010.

[50] Hunter JD (2007). Matplotlib: A 2D graphics environment. Comput Sci Eng.

[51] Halder AK, Melo A, Cordeiro MNDS (2020). A unified in silico model based on perturbation theory for assessing the genotoxicity of metal oxide nanoparticles. Chemosphere.

