# Supplementary material for: QSAR-Co-X: an open source toolkit for multitarget QSAR modelling
Source: J Cheminform. 2021 Apr 15;13:29. doi: 10.1186/s13321-021-00508-0 (PMC8048082; doi:10.1186/s13321-021-00508-0)
Supplement: Supplementary file 1 — Additional file 1. File containing the QSAR-Co-X generated ROC plots (Figures S1–3) and additional information related to the several case studies (Tables S1–5). [file 13321_2021_508_MOESM1_ESM.docx]

**QSAR-Co-X: An Open Source Toolkit for Multitarget QSAR Modelling**

Amit Kumar Halder* and M. Natália Dias Soeiro Cordeiro*

^a^LAQV@REQUIMTE/Faculty of Sciences, University of Porto, 4169-007 Porto, Portugal

***** Correspondence: [amit.halder@fc.up.pt](mailto:amit.halder@fc.up.pt), [ncordeir@fc.up.pt](mailto:ncordeir@fc.up.pt)

**List of contents:**

**Figure S1.** ROC plots for the FS-LDA and SFS-LDA models in CS1.

**Figure S2.** ROC plots for the RF and GB models in CS1.

**Figure S3.** ROC plots generated for different data-distributions in CS2.

**Table S1.** Goodness-of-fit of the linear models developed using *QSAR-Co-X* for CS1.

**Table S2.** Accuracy values of the non-linear models generated for CS1 by QSAR-Co-X.

**Table S3.** Overall performance of the final SFS-LDA linear models produced for CS2.

**Table S4.** Cut-off values used to annotate the molecules as positive in CS2 (data collected from [1]).

**Table S5.** Different cut-off values of the environmental toxicity effects of pesticides used in CS3 (data collected from [2]).


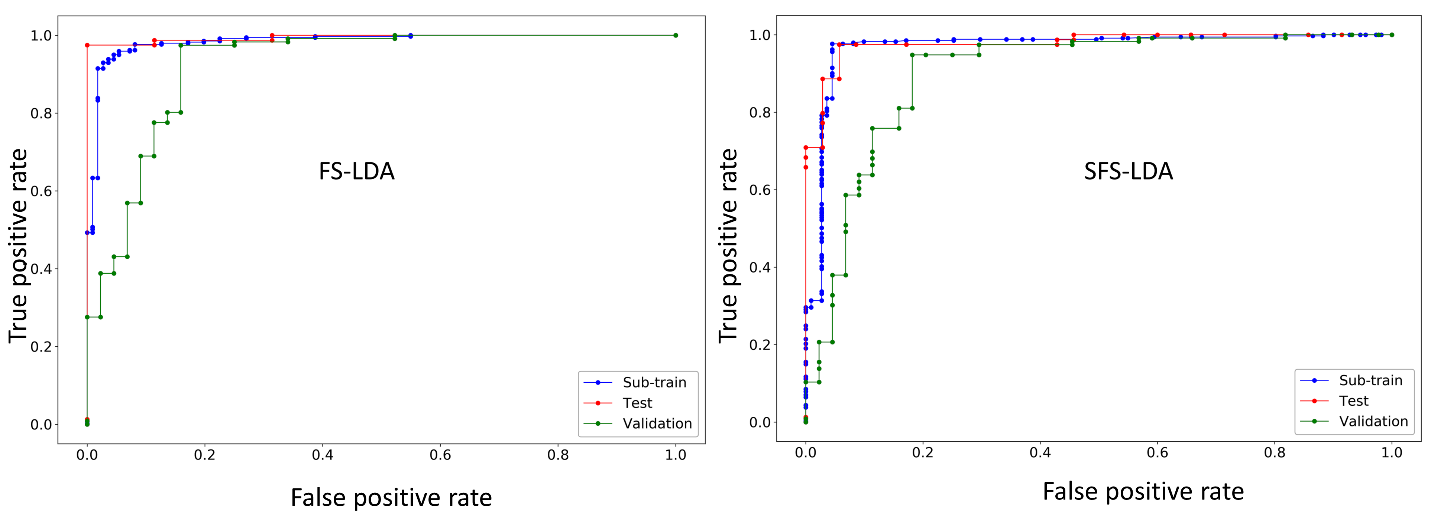


**Figure S1.** ROC plots for the FS-LDA and SFS-LDA models in CS1.

**
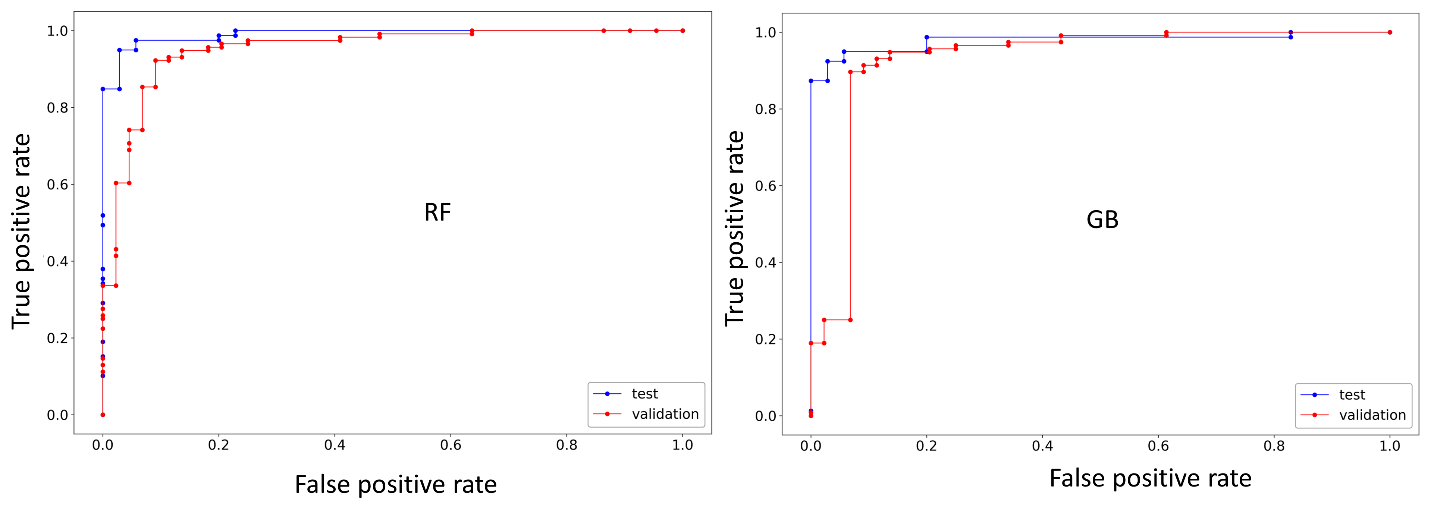
**

**Figure S2.** ROC plots for the RF and GB models in CS1.


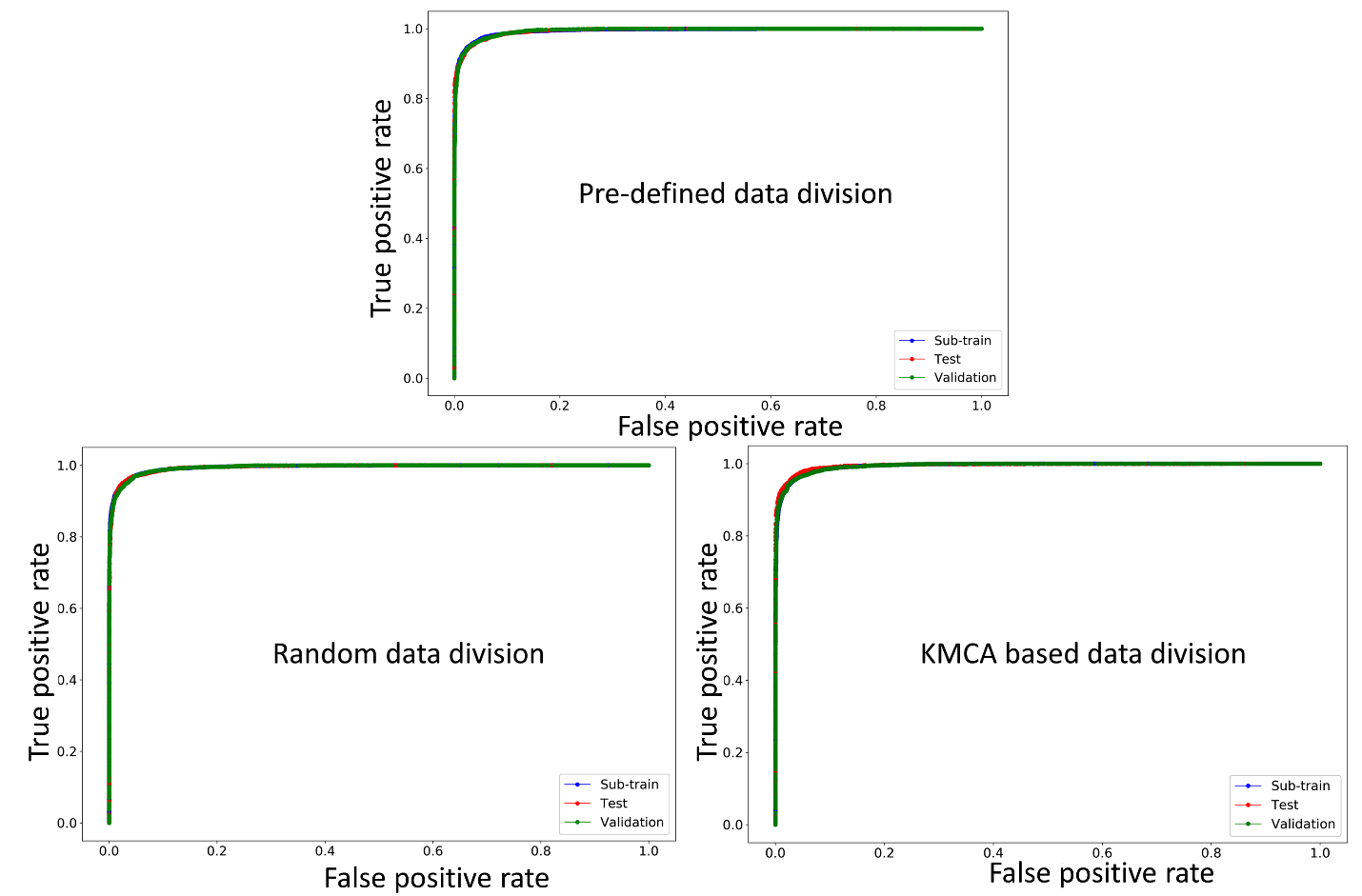


**Figure S3.** ROC plots generated for different data-distributions in CS2.

**Table S1.** Goodness-of-fit of the linear models developed using *QSAR-Co-X* for CS1.

| **Method** | **Model** | ***n*** | ***λ*** | ***p*** | ***F*** |
| --- | --- | --- | --- | --- | --- |
| FS-LDA | $\begin{matrix} IA_{i}\text{(}c_{j}\text{)}=-1.168 \Delta\text{(F06[C}-\text{O])}_{b_{t}}+7.005 \Delta\text{(VE1\_H2)}_{m_{t}}-1.894 \Delta\text{(nPyridines)}_{m_{t}} \\ -2.417 \Delta\text{[SpMax2\_Bh(s)]}_{c_{l}}-0.802 \Delta\text{(F04[C}-\text{O])}_{c_{l}}+4.116 \Delta\text{(B09} \text{[N}-\text{N])}_{c_{l}} \\ -10.270 \Delta\text{(GATS5s)}_{b_{t}}-3.067 \Delta\text{(Mor25u)}_{c_{l}}+7.969 \Delta\text{(GATS5e)}_{m_{t}} \\ +1.381 \Delta\text{(CATS3D\_09\_AA)}_{c_{l}}+8.962 \end{matrix}$ | 452 | 0.261 | < 10^−16^ | 125.00 |
| SFS-LDA | $\begin{matrix} IA_{i}\text{(}c_{j}\text{)}=-6.542 \Delta\text{[SpMin3\_Bh(s)]}_{c_{l}}-8.251 \Delta\text{(HOMA)}_{c_{l}}+3.905 \Delta\text{[nAzetdines]}_{c_{l}} \\ -4.058 \Delta\text{(B02[C}-\text{O])}_{c_{l}}-2.876 \Delta\text{(B06[O}-\text{O])}_{c_{l}}-6.319 \Delta\text{(B10} \text{[S}-\text{F])}_{c_{l}} \\ +4.125 \Delta\text{(F02[N}-\text{O])}_{c_{l}}-3.385 \Delta\text{(F02[N}-\text{O])}_{b_{t}}+1.808 \Delta\text{(CATS3D\_17\_DA)}_{c_{l}} \\ +2.049 \Delta\text{(CATS3D\_02\_AP)}_{c_{l}}+5.795 \end{matrix}$ | 452 | 0.356 | < 10^−16^ | 76.620 |

**Table S2.** Accuracy values of the non-linear models generated for CS1 by QSAR-Co-X.

| **Method** | **Parameters selected ^a^** | **Accuracy (%)** | | |
| --- | --- | --- | --- | --- |
|  |  | **Sub-training** | **Test** | **Validation** |
| RF^b^ | Bootstrap: True, Criterion: Entropy | 94.69 | 95.61 | 91.25 |
|  | Max depth: 100, Max features: Sqrt |  |  |  |
|  | Min samples leaf: 4, Min samples split: 5 |  |  |  |
|  | No estimators: 50 |  |  |  |
| *k*NN | No neighbours: 1, Weights: Uniform | 91.59 | 88.59 | 85.62 |
| SVC | C:0.1, gamma:1, Kernel: Linear | 89.16 | 88.6 | 86.87 |
| MLP **^b^** | Hidden layer sizes: 100 | 89.16 | 89.47 | 85 |
|  | Activation: Relu (Rectified linear unit function) |  |  |  |
|  | Solver: Adam (stochastic gradient based optimisation) |  |  |  |
|  | Alpha: 0.0001 |  |  |  |
|  | Learning rate: Adaptive |  |  |  |
| Bernoulli NB | Alpha: 0.1, Fit prior: False | 75.66 | 74.56 | 68.75 |
| GB^b^ | Criterion: Friedman MSE, Learning rate: 0.2  Loss: Deviance | 94.69 | 93.86 | 91.25 |
|  | Max depth:8, Max features: Sqrt, Min impurity split: None |  |  |  |
|  | Min samples leaf: 0.2, Min samples split: 0.1  No estimators: 100 |  |  |  |
|  | Subsample: 0.6 |  |  |  |

**^a^** For further details about these parameter, check the manual associated with the toolkit in <https://github.com/ncordeirfcup/QSAR-Co-X>. **^b^** Random state: ‘None’, *i.e.,* these models were generated without fixing any random state.

**Table S3.** Overall performance of the final SFS-LDA linear models produced for CS2.

| **Operator** | **Classification ^a^** | **Pre-defined data distribution** | | | **Random division** | | | ***k*MCA based division** | | |
| --- | --- | --- | --- | --- | --- | --- | --- | --- | --- | --- |
|  |  | **Str ^d^** | **Ts ^e^** | **Vd ^f^** | **Str ^d^** | **Ts ^e^** | **Vd ^f^** | **Str ^d^** | **Ts ^e^** | **Vd ^f^** |
| Method1 | TP | 11277 | 2811 | 4638 | 11141 | 2826 | 4717 | 11349 | 2807 | 4625 |
|  | TN | 15334 | 3849 | 6370 | 15463 | 3857 | 6375 | 15232 | 3837 | 6402 |
|  | FP | 705 | 160 | 295 | 652 | 144 | 242 | 722 | 185 | 335 |
|  | FN | 473 | 128 | 189 | 480 | 128 | 224 | 430 | 105 | 200 |
|  | Sn (%) | 95.6 | 96.01 | 95.57 | 95.95 | 96.38 | 96.34 | 95.47 | 95.4 | 95.03 |
|  | Sp (%) | 95.97 | 95.65 | 96.08 | 95.87 | 95.67 | 95.47 | 96.35 | 96.39 | 95.85 |
|  | Acc (%) | 95.76 | 95.85 | 95.79 | 95.92 | 96.08 | 95.97 | 95.85 | 95.82 | 95.37 |
|  | F1 score (%) | 95.03 | 95.13 | 95.04 | 95.16 | 95.41 | 95.30 | 95.17 | 95.09 | 94.53 |
|  | MCC **^b^** | 0.913 | 0.915 | 0.914 | 0.916 | 0.92 | 0.918 | 0.915 | 0.914 | 0.905 |
|  | AUROC **^c^** | 0.958 | 0.958 | 0.958 | 0.959 | 0.96 | 0.959 | 0.959 | 0.959 | 0.954 |
| Method4 | TP | 11140 | 2771 | 4565 | 11096 | 2751 | 4663 | 11124 | 2762 | 4629 |
|  | TN | 15633 | 3899 | 6505 | 15633 | 3948 | 6461 | 15533 | 3914 | 6485 |
|  | FP | 406 | 110 | 160 | 418 | 97 | 156 | 463 | 114 | 204 |
|  | FN | 610 | 168 | 262 | 589 | 139 | 278 | 613 | 144 | 244 |
|  | Sn (%) | 97.47 | 97.26 | 97.6 | 97.4 | 97.6 | 97.64 | 97.1 | 97.17 | 96.95 |
|  | Sp (%) | 94.81 | 94.28 | 94.57 | 94.96 | 95.19 | 94.37 | 94.78 | 95.04 | 94.99 |
|  | Acc (%) | 96.34 | 96.00 | 96.33 | 96.37 | 96.6 | 96.24 | 96.12 | 96.28 | 96.12 |
|  | F1 score (%) | 95.64 | 95.22 | 95.58 | 95.66 | 95.89 | 95.55 | 95.39 | 95.54 | 95.38 |
|  | MCC**^b^** | 0.925 | 0.918 | 0.925 | 0.925 | 0.93 | 0.923 | 0.92 | 0.923 | 0.92 |
|  | AUROC **^c^** | 0.961 | 0.958 | 0.961 | 0.962 | 0.964 | 0.96 | 0.959 | 0.961 | 0.96 |

**^a^** TP: True positive, TN: True negative, FP: False positive, FN: False negative, Sn: Sensitivity, Sp: Specificity, Acc: Accuracy. **^b^** Matthews correlation coefficient. **^c^** Score for the area under the receiver operating characteristic curve. **^d^** Sub-training set. **^e^** Test set. **^f^** Validation set.

**Table S4.** Cut-off values used to annotate the molecules as positive in CS2 (data collected from [1]).

| Measure of effect (Units) | Biological profile | Definition | Cutoff |
| --- | --- | --- | --- |
| MIC (nM) | Antibacterial activity | Minimum inhibitory concentration | ≤17,997.75 |
| Permeability (nm/s) | ADMET (absorption) | Permeability in cells | ≥120 |
| PPB (%) | ADMET (distribution) | Plasma protein binding | ≤75 |
| IC50 (nM)cyp | ADMET (metabolism) | Concentration that inhibits 50% of the enzymatic activity of a cytochrome | ≥5000 |
| IC50 (nM)trf | ADMET (metabolism) | Concentration that inhibits 50% of the activity of a transferase enzyme | ≥8000 |
| IC50 (nM)trp | ADMET (metabolism) | Concentration that inhibits 50% of the activity of a transport protein | ≥8000 |
| Ki (nM)trp | ADMET (metabolism) | Inhibition constant of a chemical against a transport protein | ≥8028 |
| CL_int (µL/min/106 cells) | ADMET (elimination) | Intrinsic clearance in hepatocytes | ≤6 |
| t 1/2_lm (h) | ADMET (elimination) | Half-life in liver microsomes | ≥0.5 |
| IC50 (nM)ich | ADMET (toxicity) | Inhibitory concentration at 50% against an ion channel | ≥7943.28 |
| Ki (nM)ich | ADMET (metabolism) | Inhibition constant of a chemical against an ion channel | ≥8500 |
| CC50 (nM) | ADMET (toxicity) | Cytotoxic concentration causing death to 50% of viable cells | ≥40,000 |

**Table S5.** Different cut-off values of the environmental toxicity effects of pesticides used in CS3 (data collected from [2]).

| Measure of ecotoxic effect (me) | Cutoffs^a^ | Description |
| --- | --- | --- |
| EC_25_ (mmol/ac)_TP | ≥143.54 | Concentration (mM/acre) to produce toxic effects in 25% of the terrestrial plants tested |
| EC_50_ (nM)_AP | ≥916.25 | Concentration (nM) to produce toxic effects in 50% of the aquatic plants tested |
| EC_50_ (nM)_C | ≥10632.57 | Concentration (nM) to produce toxic effects in 50% of the crustaceans tested |
| EC_50_(nM)_M | ≥3001.11 | Concentration (nM) to produce toxic effects in 50% of the molluscs tested |
| LC_50_ (nM)_Av | ≥10363739.85 | Lethal concentration (nM) to kill 50% of the birds tested according to a dietary toxicity assay |
| LC_50_(nM)_C | ≥1085.87 | Lethal concentration (nM) to kill 50% of the crustaceans tested |
| LC_50_ (nM)_F | ≥7149.9 | Lethal concentration (nM) to kill 50% of the fishes tested |
| LD_50_ (μmol/kg)_Av | ≥4147.49 | Lethal dose (mM/kg of body weight) to kill 50% of the birds tested via an acute oral toxicity assay |

**References**

1. Speck-Planche A, Cordeiro MNDS (2017) De novo computational design of compounds virtually displaying potent antibacterial activity and desirable in vitro ADMET profiles. Med Chem Res 26:2345-2356.

2. Speck-Planche A (2020) Multi-scale QSAR approach for simultaneous modeling of ecotoxic effects of pesticides, In: Roy K (ed) Ecotoxicological QSARs, Springer, New York.
