# Supplementary figures and images for: QSAR-Co-X: an open source toolkit for multitarget QSAR modelling
Source: J Cheminform. 2021 Apr 15;13:29. doi: 10.1186/s13321-021-00508-0 (PMC8048082; doi:10.1186/s13321-021-00508-0)

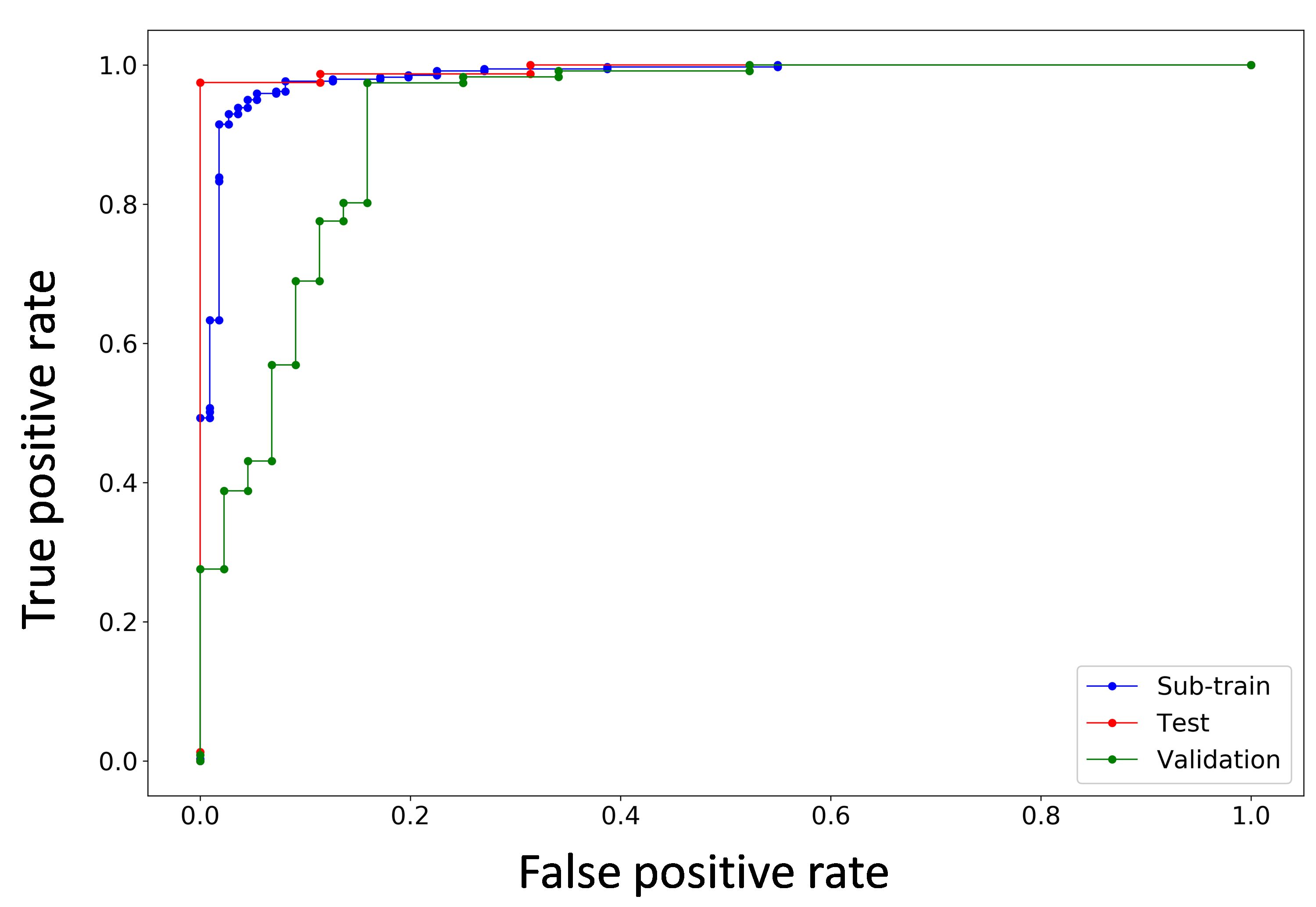

Supplement: Supplementary file 2 — Additional file 2. Folder (CS_1) containing the results (i.e., the output files from the current toolkit) of the FS-LDA, SFS-LDA, RF and GB models for case study 1. [file 13321_2021_508_MOESM2_ESM.zip › 13321_2021_508_MOESM2_ESM/CS_1/FS_LDA/pi3k_vdbj_ROC.png]

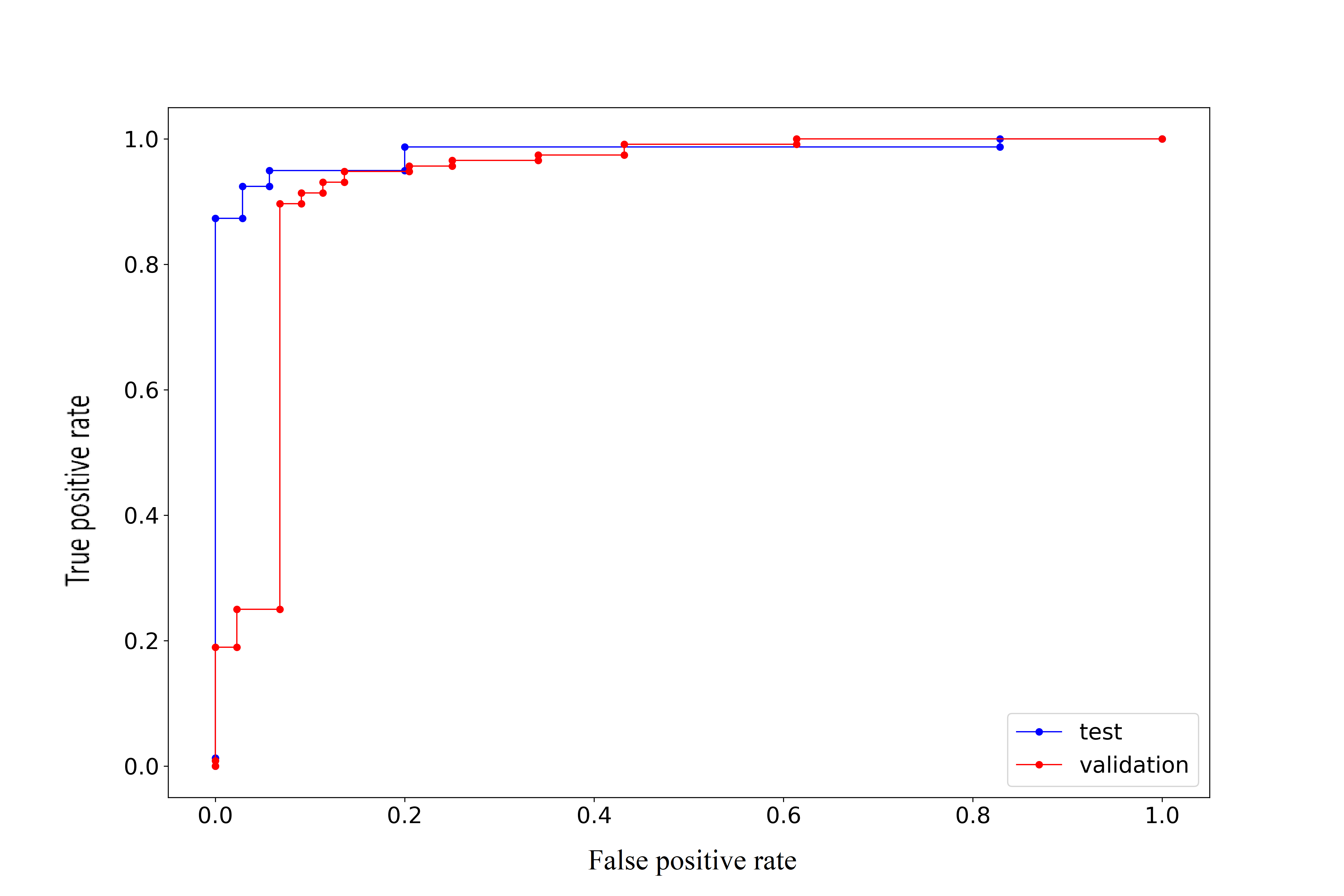

Supplement: Supplementary file 2 — Additional file 2. Folder (CS_1) containing the results (i.e., the output files from the current toolkit) of the FS-LDA, SFS-LDA, RF and GB models for case study 1. [file 13321_2021_508_MOESM2_ESM.zip › 13321_2021_508_MOESM2_ESM/CS_1/GB/pi3k_strbjg_GB_ROC.png]

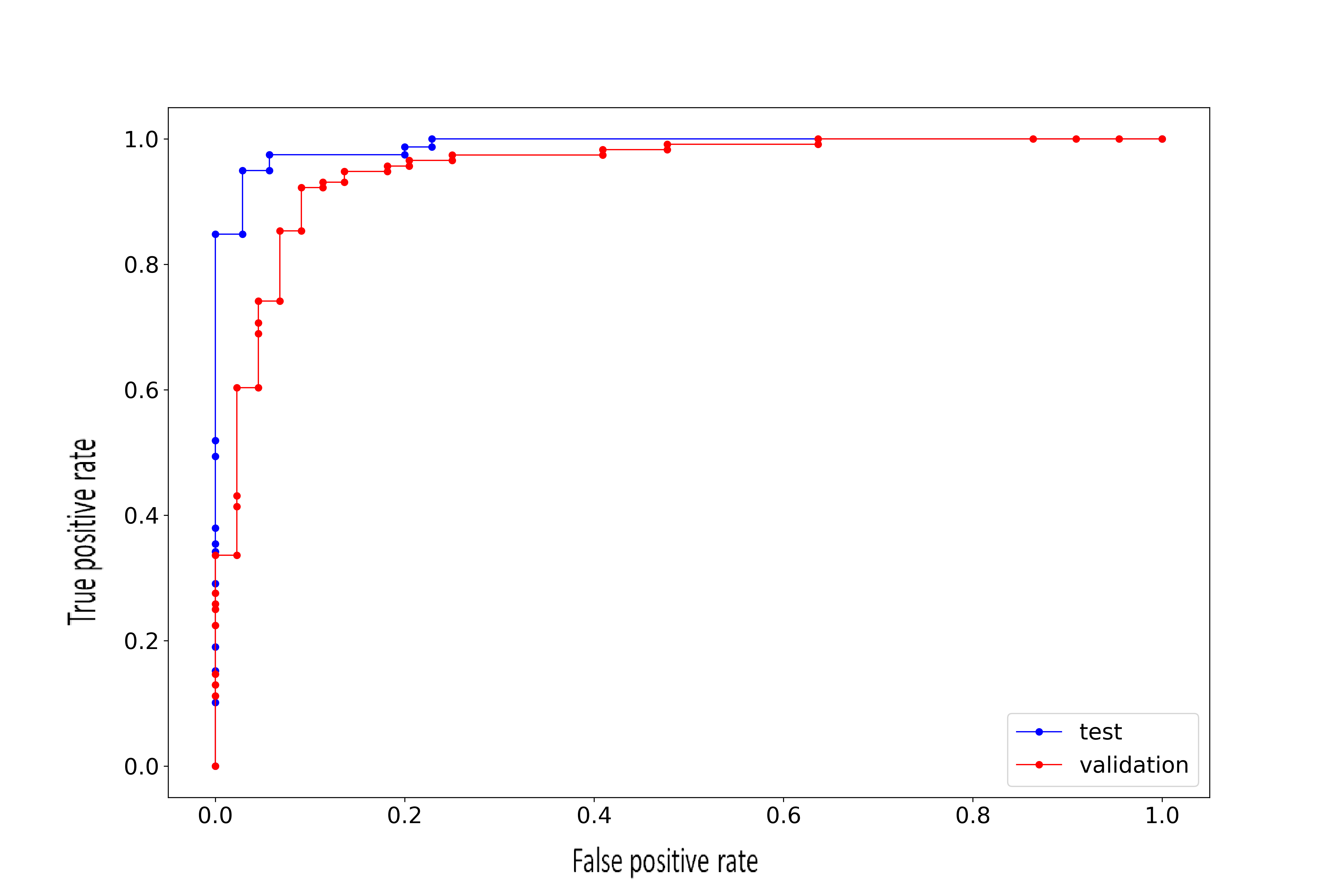

Supplement: Supplementary file 2 — Additional file 2. Folder (CS_1) containing the results (i.e., the output files from the current toolkit) of the FS-LDA, SFS-LDA, RF and GB models for case study 1. [file 13321_2021_508_MOESM2_ESM.zip › 13321_2021_508_MOESM2_ESM/CS_1/RF/pi3k_strbjg_RF_ROC.png]

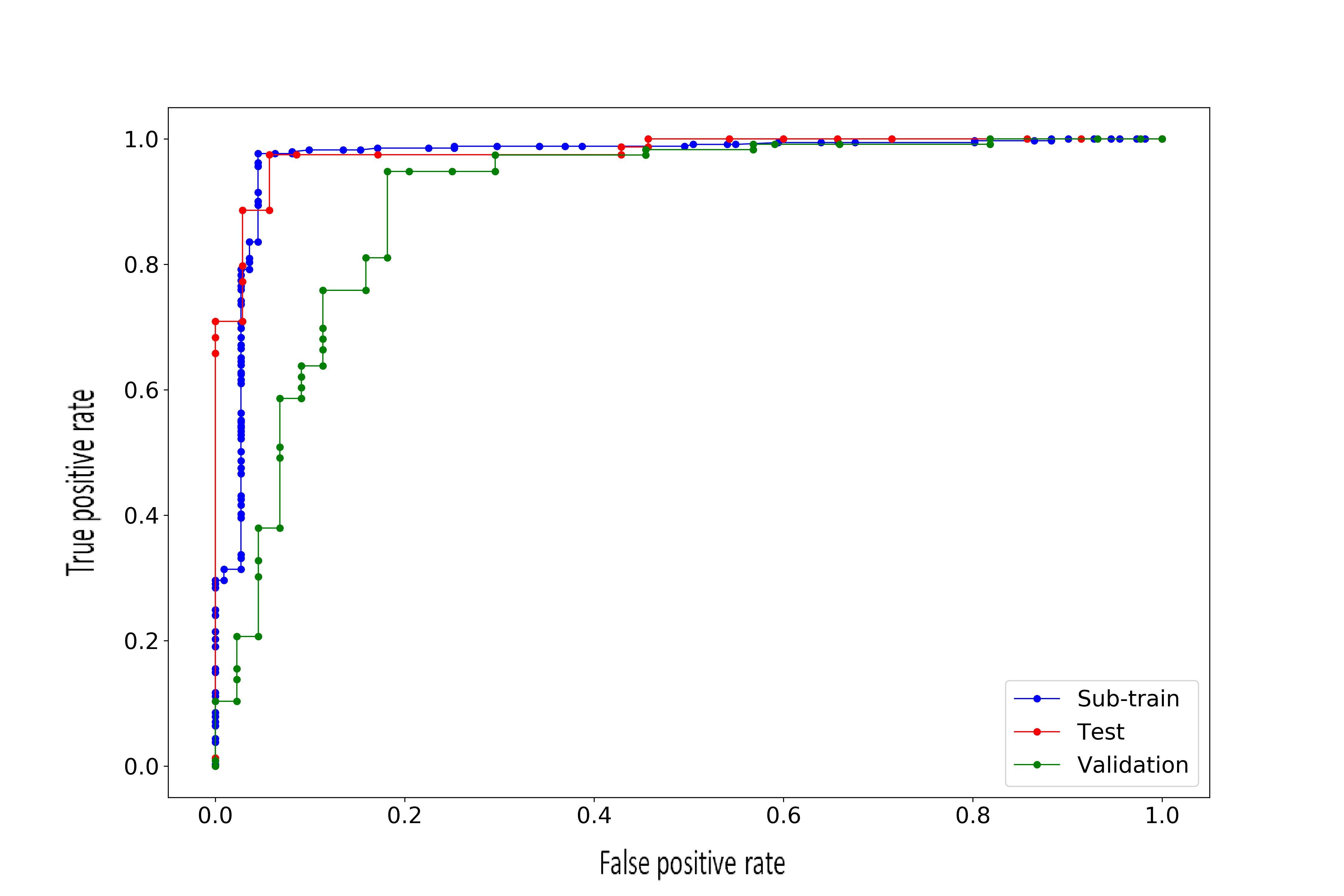

Supplement: Supplementary file 2 — Additional file 2. Folder (CS_1) containing the results (i.e., the output files from the current toolkit) of the FS-LDA, SFS-LDA, RF and GB models for case study 1. [file 13321_2021_508_MOESM2_ESM.zip › 13321_2021_508_MOESM2_ESM/CS_1/SFS_LDA/pi3k_vdbj_ROC.png]

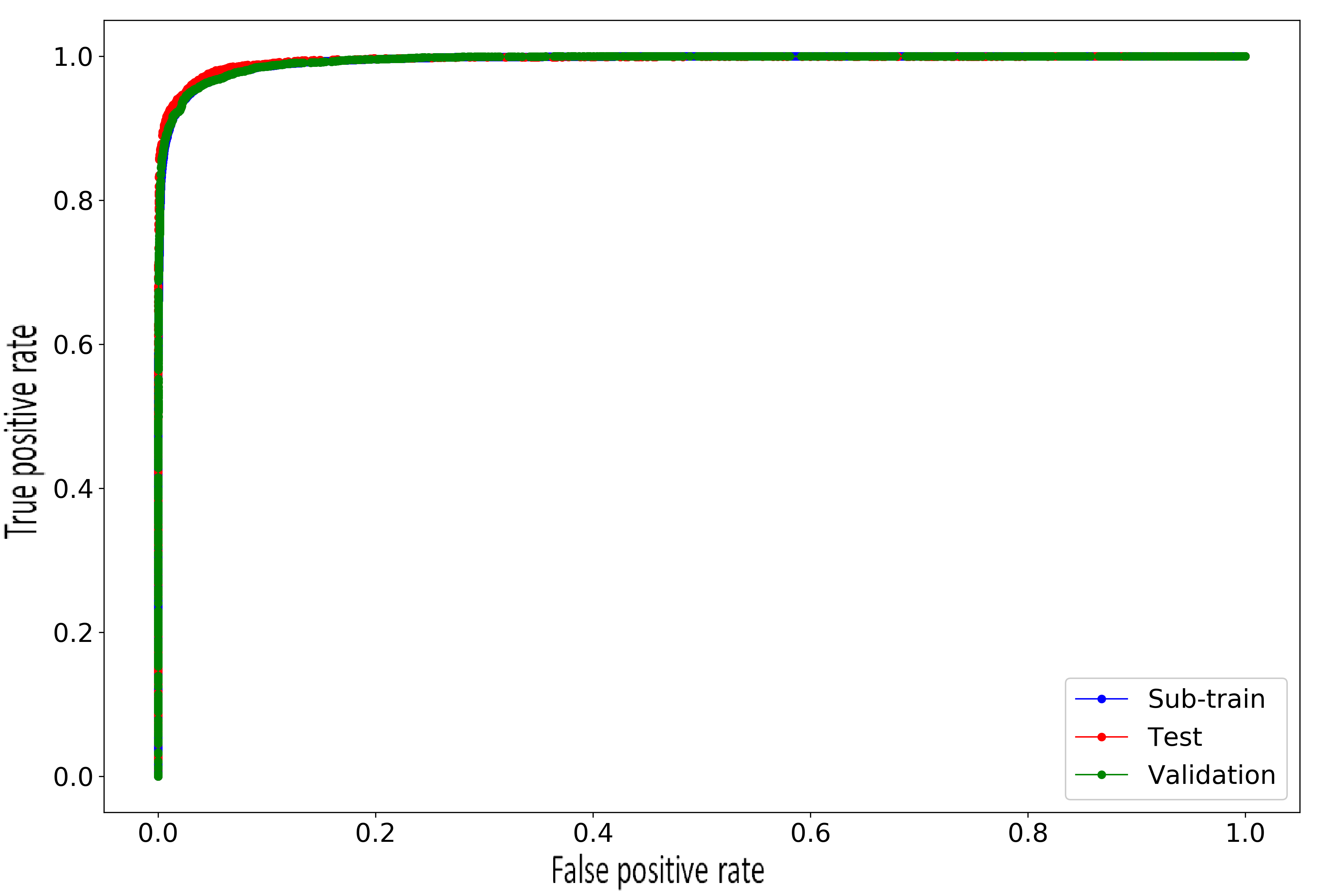

Supplement: Supplementary file 3 — Additional file 3. Folder (CS_2) containing both the input files and the results (i.e., the output files from the current toolkit) of the SFS-LDA models for Case study-2. [file 13321_2021_508_MOESM3_ESM.zip › 13321_2021_508_MOESM3_ESM/CS_2/CS2_kmca_sfslda/CS2KC_ROC.png]

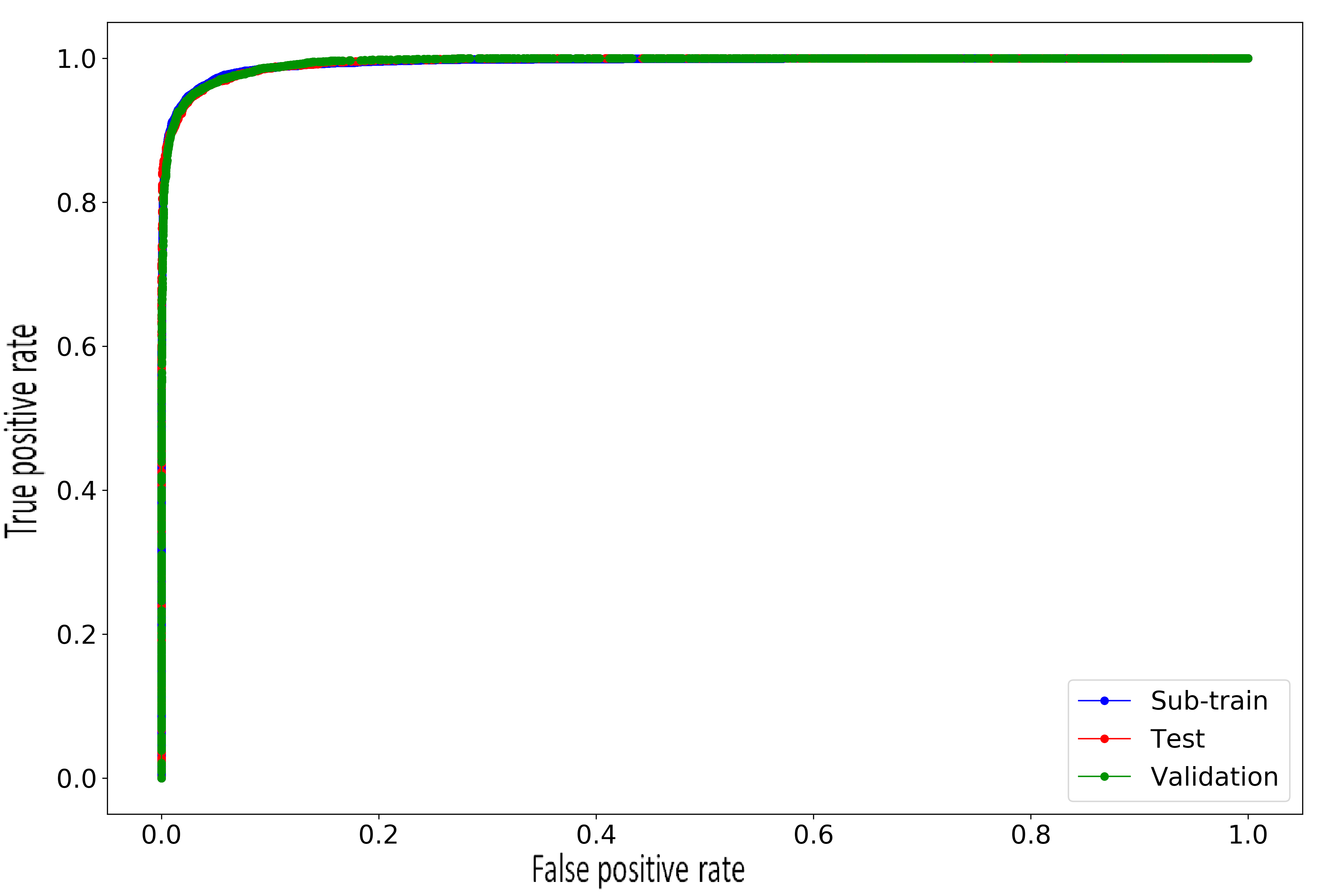

Supplement: Supplementary file 3 — Additional file 3. Folder (CS_2) containing both the input files and the results (i.e., the output files from the current toolkit) of the SFS-LDA models for Case study-2. [file 13321_2021_508_MOESM3_ESM.zip › 13321_2021_508_MOESM3_ESM/CS_2/CS2_predefined_sfslda/CS2PD_ROC.png]

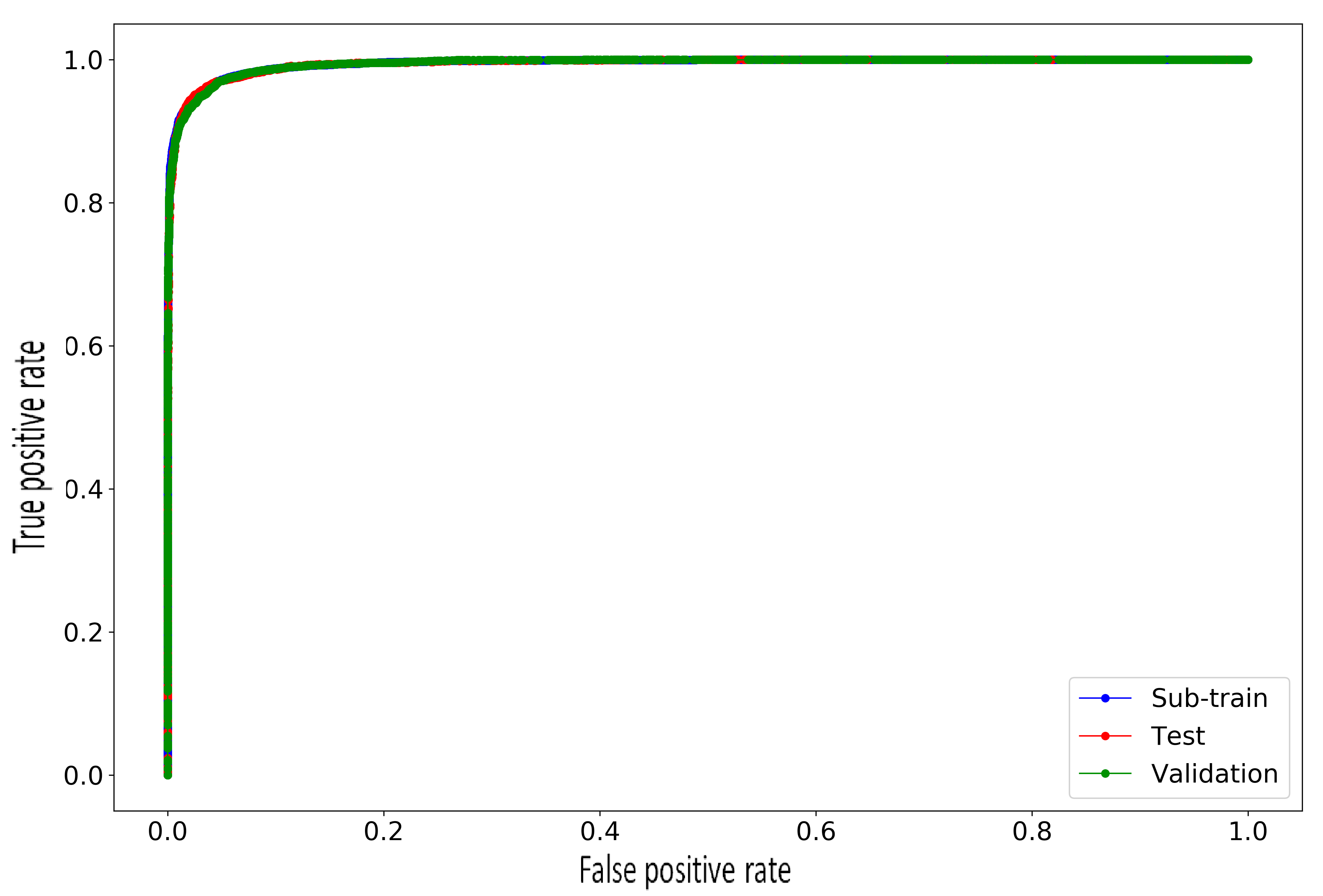

Supplement: Supplementary file 3 — Additional file 3. Folder (CS_2) containing both the input files and the results (i.e., the output files from the current toolkit) of the SFS-LDA models for Case study-2. [file 13321_2021_508_MOESM3_ESM.zip › 13321_2021_508_MOESM3_ESM/CS_2/CS2_random_sfslda/CS2RN_random_ROC.png]

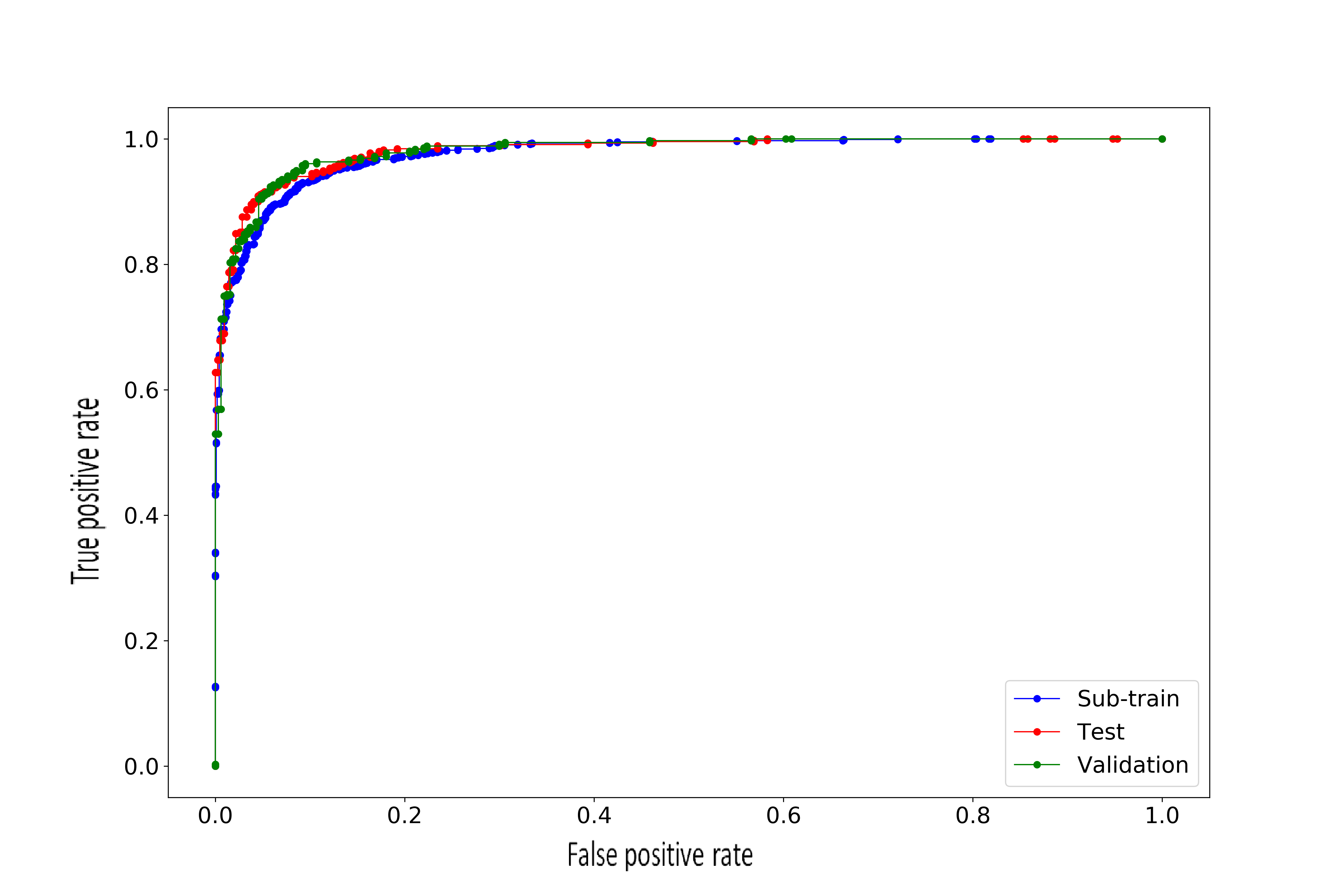

Supplement: Supplementary file 5 — Additional file 5. Folder (CS_4) containing the input file of SFS-LDA and GB models and the results (i.e., the output files from the current toolkit) obtained from the SFS-LDA for Case study-4. [file 13321_2021_508_MOESM5_ESM.zip › 13321_2021_508_MOESM5_ESM/CS_4/ROC.png]
